# Systematic genomic analysis reveals the complementary aerobic and anaerobic respiration capacities of the human gut microbiota

**DOI:** 10.3389/fmicb.2014.00674

**Published:** 2014-12-05

**Authors:** Dmitry A. Ravcheev, Ines Thiele

**Affiliations:** ^1^Luxembourg Centre for Systems Biomedicine, University of LuxembourgEsch-sur-Alzette, Luxembourg; ^2^Division 6: Comparative Genomics of Regulation System, A. A. Kharkevich Institute for Information Transmission Problems, Russian Academy of SciencesMoscow, Russia

**Keywords:** bacteria, human gut microbiome, respiration, comparative genomics, functional annotation

## Abstract

Because of the specific anatomical and physiological properties of the human intestine, a specific oxygen gradient builds up within this organ that influences the intestinal microbiota. The intestinal microbiome has been intensively studied in recent years, and certain respiratory substrates used by gut inhabiting microbes have been shown to play a crucial role in human health. Unfortunately, a systematic analysis has not been previously performed to determine the respiratory capabilities of human gut microbes (HGM). Here, we analyzed the distribution of aerobic and anaerobic respiratory reductases in 254 HGM genomes. In addition to the annotation of known enzymes, we also predicted a novel microaerobic reductase and novel thiosulfate reductase. Based on this comprehensive assessment of respiratory reductases in the HGM, we proposed a number of exchange pathways among different bacteria involved in the reduction of various nitrogen oxides. The results significantly expanded our knowledge of HGM metabolism and interactions in bacterial communities.

## Introduction

The features of the human gut create two steep oxygen gradients (Espey, 2013). A lengthwise decreasing oxygen gradient extends from the stomach to the rectum (Sheridan et al., 1990; Cooper et al., 1995; Lind Due et al., 2003), and a second oxygen gradient in the intestine is radial. The partial oxygen pressure in the submucosa zone is approximately 50% the atmospheric level. In the microvilli, the oxygen concentration decreases in a centripetal direction to approximately 10% of the atmospheric oxygen concentration at the mucus level at approximately 50–100 μm from intestine wall. A further decrease in oxygen concentration leads to nearly anoxic conditions in the lumen at approximately 1000 μm from the intestine wall (Sheridan et al., 1990; Cooper et al., 1995; He et al., 1999). The radial oxygen gradient has been shown to be associated with intestinal health. For example, in inflammatory bowel disease, the length of the radial gradient remains the same, but the partial oxygen pressure in each part of the gradient is approximately 2-fold less than in the gradient for healthy intestine (Taylor and Colgan, 2007), whereas the absorptive hyperemia shortens radial oxygen gradient and makes it more precipitous (Bohlen, 1980).

The combination of intestinal oxygen gradients and various products of food digestion produce a large variety of ecological niches for gut-inhabiting microorganisms. The radial oxygen gradient has been shown to strictly correlate with the spatial distribution of bacterial species in the human gut (Albenberg et al., 2014). In turn, human gut microbiota significantly influence oxygen gradients in the gut. Thus, oxygen-intolerant microorganisms cannot colonize the intestine if they have not been inoculated by oxygen-respiring microbes (Stewart, 2003). Based on the current knowledge of intestinal physiology, human microbiota are expected to consist of interacting and intercalating sub-communities that populate niches with specific conditions. For example, in areas proximal to the intestine wall, aerophilic microflora should predominate, whereas obligatory anaerobes should prefer the lumen space.

Although human gut microbial communities have been intensively studied in recent years (Eckburg et al., 2005; Gill et al., 2006; Sonnenburg et al., 2006; Kinross et al., 2011; Flint et al., 2012; Lozupone et al., 2012; Leimena et al., 2013; Maurice et al., 2013), almost nothing is known of the respiratory capacities of gut microbiota. In a small number of studies, mouse models have been used to analyze respiration in *Escherichia coli* (Jones et al., 2007, 2011; Spees et al., 2013) or *Salmonella enterica* (Winter et al., 2010b; Winter and Baumler, 2011; Lopez et al., 2012). Nonetheless, systematic analyses of the respiratory capacities of gut microbiota have not previously been performed *in silico*.

Investigations of the human microbiome are also hindered by the abundance of studies that have been based on the analysis of metagenomic data. Metagenome analysis is a powerful technology for determining the composition of the human gut microbiome in healthy and diseased states (Cowan et al., 2005; Kinross et al., 2011; Simon and Daniel, 2011; Cho and Blaser, 2012; Gosalbes et al., 2012; Kelly and Mulder, 2012; Walker et al., 2014). However, the exclusive analysis of metagenome data without the inclusion of completely assembled genomic sequences may exclude significant results related to the activity and metabolic potential of particular species, whereas complete sequences of bacterial genomes provide insight into the actual distribution of species and metabolic and regulatory activities of strains of microbial genomes (Koonin and Wolf, 2008; Wooley et al., 2010). A shift from the analysis of metagenome data to that of complete genome sequences is a key step in the investigation of the human gut microbial community. The number of finished and draft genome sequences for human gut inhabitants is rapidly increasing. For instance, there are 381 complete genomes for human gut microbes available through the National Institutes of Health (NIH) Human Microbiome Project (http://www.hmpdacc.org/HMRGD/).

Metagenome data and complete genome sequences for human gut microbes contain genes annotated as components of respiratory chains. However, most genomic annotations are only provided by sequence similarity analysis, which leads to a large number of misannotations (Galperin and Koonin, 1998; Schnoes et al., 2009). To minimize such misannotations, comparative genomic techniques that include genome context as additional information for gene annotation have been applied (reviewed in Osterman and Overbeek, 2003; Rodionov, 2007). This approach is based on the analysis of conserved chromosomal clusters, phyletic patterns, and transcriptional co-regulation.

## Materials and methods

From the list of human gut microbes (Nelson et al., 2010; Qin et al., 2010), we selected 250 genomes of human intestine inhabitants available in the PubSEED (Overbeek et al., 2005; Disz et al., 2010) and Integrated Microbial Genomes (IMG) systems (Markowitz et al., 2014). The following four model genomes were added to the reference set: an inhabitant of the lower gut *Escherichia coli* K-12 MG1655 (Blattner et al., 1997), intestinal inflammation causative agent *Salmonella enterica* Typhimurium LT2 (Winter et al., 2010b), model organism for the analysis of carbohydrate metabolism in the intestine *Bacteroides thetaiotaomicron* VPI-5482 (Hooper et al., 2002; Xu et al., 2003), and *Bacillus subtilis* 168, which is a model organism related to multiple gut strains. All 254 selected genomes are presented in Table S1 in the Supplementary Materials.

The PubSEED platform was used to annotate the genes for reductases. To avoid misannotations, all of the proteins with the same function were checked for orthology. Orthologs were defined as the best bidirectional hits that have similar genomic context. The search for best bidirectional hits was conducted with the BLAST algorithm (Altschul et al., 1997) implemented in PubSEED, the IMG platform (cutoff = e^−20^) and GenomeExplorer program package (Mironov et al., 2000). For the analysis of genomic context, we used PubSEED services and phylogenetic trees for protein domains in MicrobesOnline (Dehal et al., 2010).

The BLAST algorithm implemented in PubSEED and the IMG platform (cutoff = e^−10^) was additionally used to search for all of the proteins from one family. Multiple protein alignments were performed using the ClustalX v 2.0 tool (non-default parameters; protein weight matrix: BLOSUM series; gap opening: 15; gap extension: 0.15) (Larkin et al., 2007). Phylogenetic trees were constructed by the maximum-likelihood method with default parameters implemented in PhyML-3.0 (Guindon et al., 2010) and visualized using the interactive viewer Dendroscope (Huson et al., 2007). To predict specificities according to the specificity-determining positions (SDP), the SDPfox web tool (Mazin et al., 2010) was used (the maximum percent of gaps allowed in a group in each column = 50%). To predict protein subcellular locations, the CELLO (Yu et al., 2006) web tool was used.

All of the annotated genes are represented as a subsystem in PubSEED (http://pubseed.theseed.org//SubsysEditor.cgi?page=ShowSpreadsheet&subsystem=Respiration_HGM), and all of the protein sequences for the annotated genes in FASTA format are represented in file Sequences S2 in the Supplementary Materials.

## Results and discussion

In this study, we aimed to investigate the respiratory capacities of the human gut microbiome. To identify respiratory genes in microbial genomes, we applied a set of comparative genomics and genome context-based techniques to accurately annotate the respiratory reductases. This study included an analysis of 254 complete and fragmentary microbial genomes that were selected based on the following criteria: analyzed genomes should be assigned as having occurred in human fecal samples in previous studies (Nelson et al., 2010; Qin et al., 2010); genome sequences should be available in the GenBank database (Benson et al., 2013); and genomes should be available for analysis in both the PubSEED (Overbeek et al., 2005; Disz et al., 2010) and IMG (Markowitz et al., 2014) systems. The presence of genomes in both systems was dictated by the different opportunities provided by each of these systems in conjunction with our multi-approach analysis of respiration in human gut microbiota.

### Analysis of the reference set of genomes

The analysis of metabolic capacities in incomplete genomes generates a level of uncertainty. In the analysis of complete genomes, studied genes are either present or absent; however, in the case of an incomplete genome, an analyzed gene can be marked as absent if the gene does not occur in the genome or if it cannot be detected because of the incomplete genome sequence. Of the 254 reference genomes used in this work, only 55 genomes (22%) had a finished status and were definitely complete (Table S1 in Supplementary Materials). The remaining 199 genomes had a draft status and were incompletely assembled. Unfortunately, no information on the completeness of the available sequence for the 199 draft genomes was provided in any database. Therefore, to estimate the completeness of draft genomes, we applied two different approaches (Table S1 in Supplementary Materials).

The first approach to estimating genome completeness was to analyze the essential genes found in a certain genome sequence (Dupont et al., 2012; Albertsen et al., 2013). Unfortunately, this approach was not successful. The lowest percentage of essential genes (84.9%) was observed for the genome of *Gordonibacter pamelaeae* 7-10-1-bT (Table S1 in Supplementary Materials), which had a finished status and was definitely complete and could not be used to determine a reasonable threshold for distinguishing between complete and incomplete genomes. Therefore, we used a new approach based on a comparison of genome lengths. The size of each of the 199 draft genomes was compared with the size of closely related finished genomes. Closely related genomes were defined as genomes belonging to the same species or genus. When no genomes of the same species/genus were available, the closest related finished genomes were detected based on 16S rRNA similarity. With the sequence of 16S rRNA as a query, the search for the most similar sequences was provided by a nucleotide BLAST search in the National Center for Biotechnology Information (NCBI) reference genomic sequence database. A genome was considered to be complete when its length was greater than at least one of the finished closely related genomes. Otherwise, the genome was considered to be incomplete. Ultimately, 149 (58.7%) of 254 genomes were assigned as probably complete and 50 were assigned as incomplete (19.7%, Table S1 in Supplementary Materials).

### Annotation of genes for aerobic respiration

Across the steeply dissipating oxygen gradient in the intestinal lumen, representatives of the human gut microbiome should demonstrate various capacities in their utilization of molecular oxygen. To assess these different capacities, we studied the distribution of aerobic reductases among the reference set of genomes. Two types of known aerobic reductases can be distinguished (Table S2 in Supplementary Materials). The first type includes cytochrome *bd* reductases, which have a high affinity to molecular oxygen and are usually expressed under low oxygen concentrations. In certain organisms, these reductases participate in both respiration and oxygen defense (Moshiri et al., 1991; Dincturk et al., 2011), and reductases of this type are necessary when there are micro- or nanomolar concentrations of oxygen (Baughn and Malamy, 2004). We deemed these reductases as “microaerobic.” The second type of reductases includes enzymes with various cytochromes (Table S2 in Supplementary Materials). Such enzymes are usually expressed under high oxygen levels; consequently, we deemed these reductases “aerobic” reductases. Microaerobic reductases were found in 132 of the 254 reference genomes (51.9%). In 29 of the 254 references genomes (11.4%), we found aerobic reductases (Table S1 in Supplementary Materials) that are orthologs of the four known enzymes, Cyo, Qox, Cta, and Cco. Although aerobic reductases include different types of heme molecules, the largest subunits of these complexes (CyoB, QoxB, CtaD, and CcoN) share a sequence similarity and belong to the same protein family (Pfam ID: PF00115). Thus, we searched for homologs of these proteins to predict novel candidate aerobic reductases. However, no new representatives of this family could be identified in the 254 reference genomes.

A novel type of microaerobic respiratory reductase was predicted in this research for the gram-positive bacterium *Faecalibacterium prausnitzii* A2-165, which represents 5–15% of the total bacterial population and is thus the most abundant bacterium in the healthy gut (Li et al., 2008). Among human intestine microbiota, *F. prausnitzii* is one of the most abundant producers of butyrate (Flint et al., 2012), a short-chain fatty acid with anti-inflammatory properties that serves as the main energy source for colonocytes (Tremaroli and Backhed, 2012). *F. prausnitzii* was also shown to be closely associated with healthy and diseased states and can be used as an indicator of intestinal health in adult individuals (reviewed in Miquel et al., 2013).

**Figure 1:**
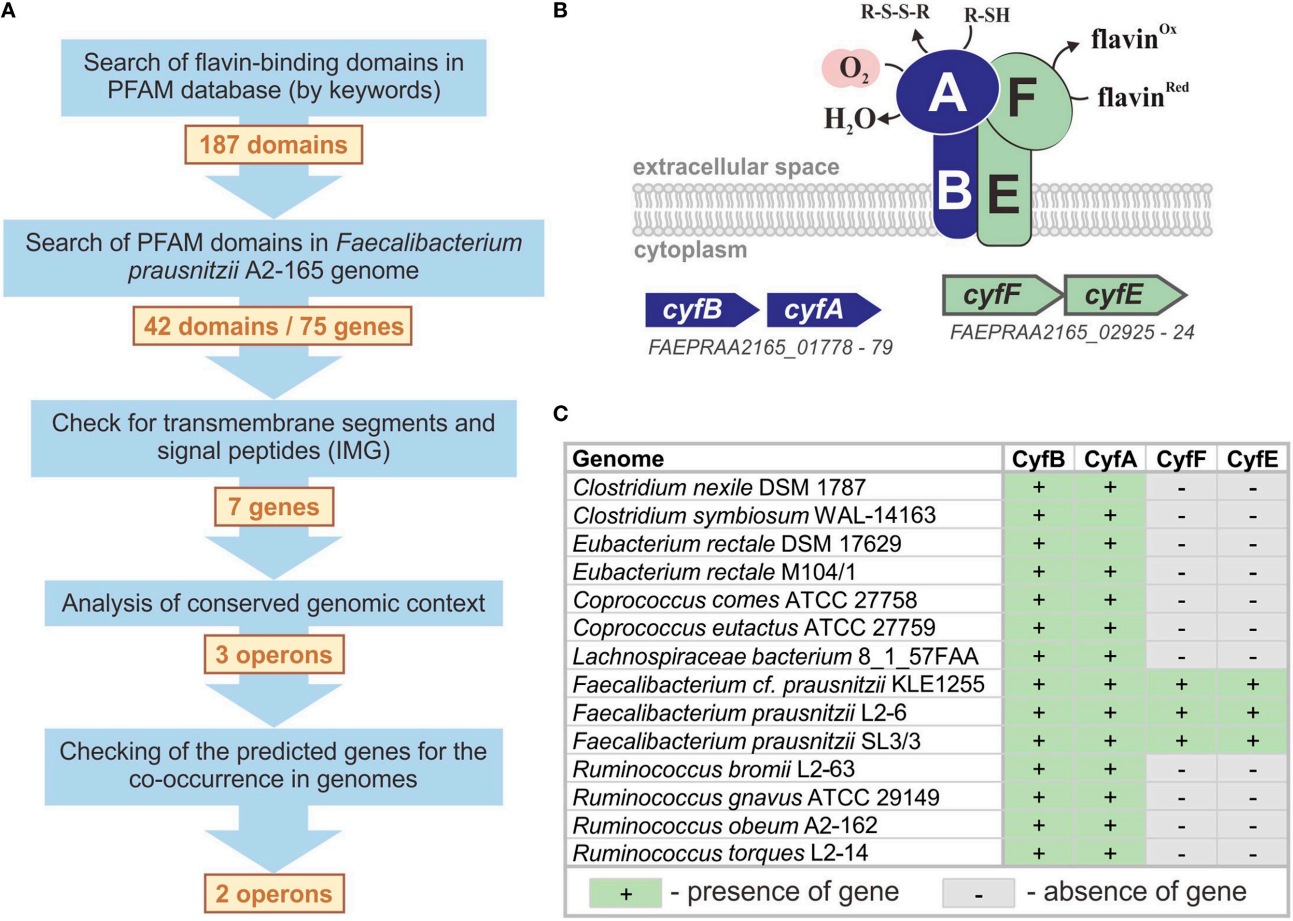
**Prediction of the novel microaerobic reductase. (A)** The workflow for the prediction of the microaerobic reductase in *F. prausnitzii*. **(B)** The model for a flavin- and thiol-dependent extracellular microaerobic reductase Cyf in *F. prausnitzii*. **(C)** Distribution of *cyf* genes in reference genomes.

*F. prausnitzii* is particularly important for studies of respiration. Although it is an extremely oxygen-sensitive organism, *F. prausnitzii* can grow under microaerobic conditions (Khan et al., 2012b). Another oxygen-sensing organism capable of microaerobic growth is *Bacteroides fragilis*. During microaerobic growth, this bacterium uses microaerobic reductase CydAB (Baughn and Malamy, 2004). Nevertheless, the analysis of respiratory reductases conducted in this research demonstrated that the *F. prausnitzii* genome lacks the genes for this reductase. However, the potential for microaerobic growth of *F. prausnitzii* has been reported in the presence of extracellular flavins and thiols. Thus, *F. prausnitzii* has been predicted to use a respiratory extracellular electron shuttle; however, the responsible gene or protein has not been identified (Khan et al., 2012a,b). Therefore, we searched for candidate genes that could encode a flavin- and thiol-dependent extracellular microaerobic reductase. Based on previous data, we concluded that candidate genes should satisfy the following requirements: (1) presence of flavin- and thiol-binding domains, (2) presence of signal peptides and/or transmembrane segments indicating their extracellular and/or membrane localization, respectively, and (3) appropriate genome context, i.e., co-localization with candidate oxygen-binding proteins. We used a step-by-step workflow to predict *F. prausnitzii* A2-165 proteins that correspond to all of these requirements (Figure 1A).

The search by keywords in the Pfam database (Finn et al., 2014) revealed 187 domain families capable of binding flavins or interacting with flavin-binding proteins. Of these 187 Pfam families, 145 had no sequence similarities in the studied genome. For the other 42 Pfam families, 75 were candidate proteins encoded in the *F. prausnitzii* A2-165 genome; however, only 7 of the 75 candidate proteins included the predicted signal peptide and/or transmembrane segments, and only 3 of the 7 candidate genes had the correct genomic context, i.e., were located in candidate operons (Table S3 in Supplementary Materials). The operon *FAEPRAA2165_01778-79* is particularly important because of the presence of a gene of the AhpC/TSA family, which includes proteins that bind oxygen and thiols (Chae et al., 1994). Because the product of this gene should bind thiols and oxygen, the operon *FAEPRAA2165_01778-79* is an ideal candidate for the desired system. Another gene in this operon encodes a transmembrane FeS-cluster protein that belongs to the family PF01077. Proteins of this family can interact with flavin-binding proteins but do not bind flavins themselves (Crane et al., 1995, 1997). Thus, additional genes (and operons) are required to complete the flavin-dependent microaerobic system in *F. prausnitzii* A2-165, and the two identified operons are good candidates for such a role. For a more precise prediction of the flavin-binding component, we analyzed the co-occurrence of the three candidate operons in the genomes of five *F. prausnitzii* strains (Table S4 in Supplementary Materials). Only the *FAEPRAA2165_01778-79* and *FAEPRAA2165_02925-24* operons were found in all five studied genomes, so we excluded the *FAEPRAA2165_02240-42* operon from further analysis (Figure 1A). The genes *FAEPRAA2165_01778* and *FAEPRAA2165_02925* (referred to as *cyfB* and *cyfF*, respectively) are membrane electron-transfer proteins. The gene *FAEPRAA2165_01779* (referred to as *cyfA*) is an extracellular oxygen- and thiol-binding protein, whereas *FAEPRAA2165_02924* (referred to as *cyfF*) is an extracellular flavin-binding protein (Figure 1B).

An analysis of the reference genomes revealed that Cyf reductase is present in 14 genomes (Table S1 in Supplementary Materials). Nevertheless, only the *cyfAB* operon was found in all genomes, whereas the presence of *cyfEF* appeared to be a specific feature of the three reference *Faecalibacterium* spp. genomes (Figure 1C). The absence of *cyfEF* in the other 11 genomes can be explained in two ways: (1) another flavin-binding component is used in the Cyf system in these organisms or (2) the extracellular reduction of oxygen is independent of flavins in these organisms. Because *F. prausnitzii* A2-165 flavin-dependent respiration has only been previously observed under microaerobic conditions (Khan et al., 2012a,b), we identified the Cyf reductase as microaerobic.

### Annotation of genes for anaerobic respiration

To define the repertoire of electron acceptors utilized by the reference set of genomes, we identified genes for all known anaerobic reductases of electron acceptors (Table S2) except for the reductases of metal and organohalide respiration. Overall, we evaluated 32 types of anaerobic reductases, and 26 were found in the reference genomes (Table S1 in Supplementary Materials). These enzymes are capable of reducing electron acceptors, such as tetrathionate, thiosulfate, polysulfide, sulfite, adenylyl sulfate, heterodisulfides, fumarate, trimethylamine N-oxide (TMAO), dimethyl sulfoxide (DMSO), nitrate, nitrate, nitric oxide, nitrous oxide, selenate, and arsenate; however, genes for the reduction of ethylbenzene, chlorate, and perchlorate have not been found in the reference genomes. For certain electron acceptors, more than one type of reductase was described in the literature (Table S2 in Supplementary Materials), although multiple types of such redundant reductases were not always found in the reference genomes. For example, two types of selenate reductase, Ynf and Ser, were previously described (Schroder et al., 1997; Guymer et al., 2009), but only one Ynf reductase was detected in the reference genomes. Moreover, we did not find the denitrifying nitrite reductase NirSTBM, DMSO/TMAO reductase Ddh, arsenate reductase Aio, or heterodisulfide reductase HdrEF in the reference genomes.

A large number of anaerobic reductases contain subunits belonging to the molybdopterin oxidoreductase family (Pfam: PF00384). These enzymatic components of reductases bind and reduce electron acceptors and determine the specificity of the entire reductase (Igarashi et al., 1997; Czjzek et al., 1998; Dias et al., 1999; Bender et al., 2005; Schwarz et al., 2009). In addition to respiratory anaerobic reductases (Table S2 in Supplementary Materials), this family also includes assimilatory nitrate reductases (Lin et al., 1993; Nakano et al., 1995; Aichi et al., 2001), cytoplasmic biotin sulfoxide reductases (Pierson and Campbell, 1990; Pollock and Barber, 1995), and formate dehydrogenases (Heider and Bock, 1992; Wang and Gunsalus, 2003). In total, 40 proteins from the PF00384 family were previously experimentally analyzed (Table S2 in Supplementary Materials). To improve the functional annotations of respiratory reductases, we carefully analyzed all of the proteins of the PF00384 family in the reference genomes.

**Figure 2:**
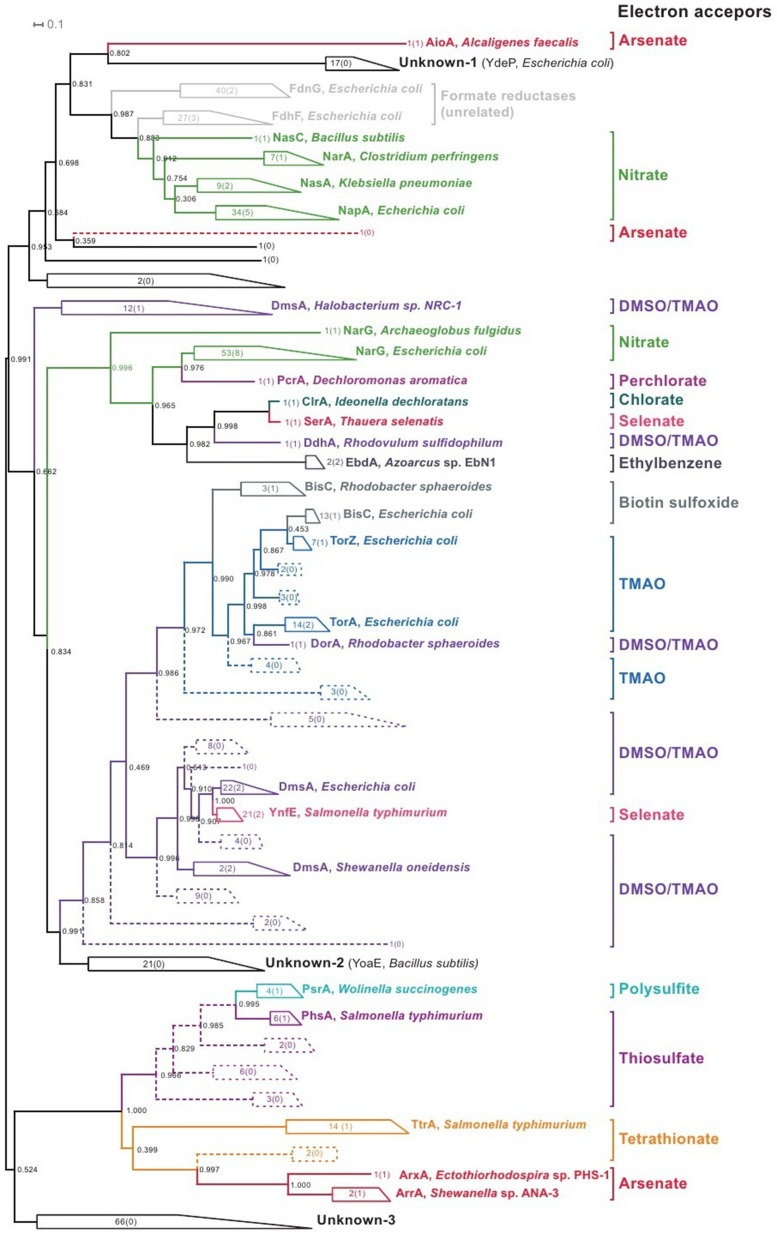
**Phylogenetic tree for the proteins of PF00384 family**. All of the monophyletic groups with conserved specificities are collapsed (for the uncollapsed tree, refer to Figure S1 in Supplementary Materials); the figures show the number of proteins in each branch, and the figures in brackets show the number of known proteins in each branch, with one example of known proteins shown for each branch. Braches with specificities identified by an analysis of the phylogenetic tree are solid; branches with specificities identified by SDP analysis are dashed.

In addition to known proteins, the reference genomes contain 440 proteins for PF00384 family (Sequences S1 in Supplementary Materials). An analysis of the phylogenetic tree for this family (Figure 2, Figure S1 in Supplementary Materials) revealed a number of monophyletic branches that included known proteins. For proteins in such branches, we analyzed the genomic context for the genes coding PF00384 proteins. When the genomic context was conserved within a branch, the specificity of known proteins was extrapolated to all of the proteins in the branch. Additionally, for further extrapolation of known specificities, we used the specificity-determination positions (SDP) method (Kalinina et al., 2004; Mazin et al., 2010) to predict novel reductases specific to DMSO/TMAO, TMAO, thiosulfate, tetrathionate, and arsenate. These predicted proteins did not form monophyletic branches with the known proteins (Figure 2) and could not be identified by an analysis of the phylogenetic tree. Using initial phylogenetic-based extrapolation, we predicted specificities for 191 proteins, and after applying the SDP approach, we predicted specificities for an additional 48 proteins.

For the 109 proteins of the PF00384 family, specificities could not be predicted by phylogenetic analysis or the SDP approach, and an analysis of the genomic context of these proteins was also unsuccessful. Most of these proteins belong to three monophyletic branches (Figure 2, groups labeled as “Unknown”). The first branch contains cytoplasmic proteins, including the *E. coli* protein YdeP, which does not have a sufficiently clear function (Masuda and Church, 2003). The second branch includes two cytoplasmic proteins from *B. subtilis*, YoaE, and YyaE, which were annotated as formate dehydrogenases, although their position in the PF00384 family phylogenetic tree indicates that these proteins could be involved in the reduction of derivatives of sulfur or nitrogen oxides, such as Dms, Tor, and BisC proteins. The third branch includes 66 proteins of unknown function, and most were found in the genomes of *Eggerthella* sp. 1_3_56FAA, *Gordonibacter pamelaeae* 7-10-1-bT, and *Clostridium* spp. These proteins are distant from all previously described proteins, and their specificity cannot currently be identified using comparative genomics methods. Additionally, all of these proteins are chromosomally co-localized with multiple proteins that are characteristic for respiratory reductases, such as iron-sulfur cluster proteins, membrane anchor subunits, and TorD family chaperones. Thus, this branch contains the predicted anaerobic respiration reductases with unknown specificities.

In addition to the expansion of reductase specificities to homologous proteins, we also predicted one thiosulfate reductase that has no known homologs with the same specificity. In the genome of *Burkholderiales bacterium* 1_1_47, we found a putative operon containing homologs of certain *nos* (nitrous oxide reductase) genes; however, the gene for the catalytic subunit *nosZ* was not found in this genome, although the detected putative operon also contained the gene for the cytochrome *c-552/4*-like protein. Because *c*-type cytochromes are often involved in anaerobic respiration (Richardson, 2000), we propose that this protein can act as a catalytic subunit. The orthologs of this operon were found in the genomes of *Shewanella* spp., including *Shewanella oneidensis* MR-1. In *S. oneidensis*, this operon was previously shown to be strongly induced by the presence of thiosulfate (Beliaev et al., 2005). Thus, we predicted that the operon found in *B. bacterium* 1_1_47 (locus tags: HMPREF0189_01800-01791) encodes a novel thiosulfate reductase, and the genes of this operon were renamed *tsrABCDEFGHIJ*.

### Relation of the reference genomes to oxygen

Based on the distribution of aerobic, microaerobic, and anaerobic reductases, we classified all of the reference genomes as aerophilic, microaerophilic, or anaerobic, respectively (Table S1 in Supplementary Materials). It should be noted that this classification is only reliable for finished and most likely complete genomes because in incomplete genomes, we cannot distinguish between the actual absence of the analyzed genes and absence of the region that contains these genes. Among the 204 finished and probably complete genomes, 25 were identified as aerophilic because of the presence of aerobic-type reductases. Notably, all of the aerophilic genomes except for *Helicobacter bilis* ATCC 43879 contain microaerophilic reductases. Consequently, all of the analyzed aerobic organisms are most likely capable of living in different parts of the intestine under both oxygen-rich and oxygen-limited conditions. Another 92 finished and probably complete genomes were defined as microaerophilic, which indicated that they included microaerobic but not aerobic reductases. Remarkably, 27 of these microorganisms should be strong microaerobes because they include microaerobic reductases and are absent anaerobic reductases. The residual 87 finished and probably complete genomes were defined as anaerobic because of the absence of either microaerobic or aerobic reductases. For incomplete genomes, we confidently identified 4 genomes as aerophilic because of the presence of genes for aerobic reductases. Microaerobic reductases were detected in 19 incomplete genomes; therefore, these genomes were classified as at least microaerophilic because we cannot to be sure about the absence of aerobic reductases in these organisms. No reductases were found in 27 incomplete genomes; therefore, these organisms were classified as most likely anaerobic (Table S1 in Supplementary Materials).

To validate our predictions of the oxygen utilization capabilities of the reference genomes, we compared our predictions to data from the IMG database (Markowitz et al., 2014). The IMG system contains two types of data on the methods by which an organism uses oxygen, and these data are referred to as metadata and phenotypes/metabolism from pathway assertions. The metadata are usually from the annotations to the genome sequence, and their primary source is often vague. The phenotypes/metabolism from pathway assertions are based on genomic analyses and the presence of certain functional categories of genes in the reference genome. Among 254 analyzed reference genomes, both types of data were available for 98 genomes; however, only metadata were available for 136 genomes, only phenotypes/metabolism from pathway assertion data were available for 5 genomes, and no data were available for 15 genomes (Table S1 in Supplementary Materials). The first group of genomes that included both types of available data indicated 42 cases of inconsistency between the metadata and pathway assertion data. However, for all 98 genomes, our predictions were consistent with the pathway assertion data. Thus, our genome-based predictions in the use of oxygen are consistent with the IMG metadata for 56 genomes (57.1%) and inconsistent for 42 genomes (42.9%). For the second group that only included the available metadata, our results were consistent with the IMG metadata for 122 genomes (89.7%), whereas for 14 genomes (11.3%), inconsistencies were detected between our results and the IMG metadata. For the third group of genomes that only included the available pathway assertion data, our results were consistent with the IMG data for all 5 genomes. Thus, our predictions were consistent with all of the phenotypes/metabolism from pathway assertion data, whereas a comparison of the predictions with the metadata resulted in a consistency and inconsistency of 76.1 and 23.9%, respectively.

The results of our genomic analysis were always consistent with data derived from pathway assertion but often inconsistent with the metadata. All 42 cases of inconsistencies between the different types of IMG data include genomes that are assigned as anaerobes by the metadata but as aerobes by the pathway assertion data and our results. Such inconsistency can be attributed to a lack of comprehensive experimental data on the lifestyle of the reference organisms. Among these 42 organisms, experimental data were only available for *Bacteroides fragilis*, being considered an obligatory anaerobe until the identification its ability to live in a nanoaerobic environment because of the presence of Cyd reductase (Baughn and Malamy, 2004). The genes for Cyd reductase were identified in all 42 inconsistent genomes.

In the group that included 14 inconsistent genomes where only metadata were available, all of the genomes were predicted to be anaerobes, whereas the IMG data assigned them as aerobic, facultative aerobic, or microaerophilic. Four of these genomes, *Escherichia* sp. 1_1_43, *Streptococcus* sp. 2_1_36FAA, *Streptococcus infantarius* ATCC BAA-102, and *Lactobacillus amylolyticus* DSM 11664, were incomplete; therefore, the observed inconsistencies can be explained by the absence of regions with genes for aerobic/microaerobic reductases in the current version of the genome. The inconsistencies for the remaining 10 genomes required additional analysis, and all were finished or most likely complete and classified by us as anaerobic.

Recently, the spatial distribution of gut microbiota was correlated with the radial oxygen gradient (Albenberg et al., 2014). In this research, the authors compared the microbial spatial distribution in the gut to data in the literature on the oxygen tolerance of various microorganisms. Because data are not available in the literature for most human gut microbes, the authors used data for related species and strains belonging to the same genera as the analyzed gut microorganism. An analysis of the literature revealed data for 65 genera that included 233 of the studied genomes (Table S1 in Supplementary Materials). For 211 (85.8%) genomes, the current research was consistent with data from the literature, whereas the results for 35 (14.2%) genomes were not inconsistent. The first group of inconsistencies included 29 genomes that were predicted by us to be microaerobic but were assigned as anaerobic in the analyzed literature. All of these genomes contain the microaerobic reductases Cyd or Cyf. Thus, this inconsistency may have resulted from an excessively straightforward classification of microorganisms. In most of the analyzed literature, the studied microorganisms were evaluated for growth only under oxygen concentrations close to atmospheric but not under microaerobic conditions. The examples of *B. fragilis* and *F*. *prausnitzii* confirmed that bacteria assessed as strong anaerobes can also utilize oxygen in micromolar and nanomolar concentrations (Baughn and Malamy, 2004; Khan et al., 2012b).

Another type of inconsistency between our results and the data from the literature was observed for six genomes assigned as facultative anaerobes or microaerophiles. Among this group, we assigned four genomes as most likely anaerobic. These genomes were predicted to be incomplete, and most of the genes for aerobic and/or microaerobic reductases will be found eventually in genome regions that are not currently available. The remaining two genomes belong to the species *Pediococcus acidilactici* (strains 7_4 and DSM 20284). Both of these genomes were predicted to be probably complete, but no aerobic or microaerobic reductases were found; nonetheless, this species was shown to be aerotolerant (Rodriguez-Palacios et al., 2009). Two explanations of this conflict are possible: (1) experimental data for oxygen tolerance consider that *P. acidilactici* is from bovine intestine, and human gut strains possibly lack this tolerance, or (2) *P. acidilactici* contains an unknown aerobic/microaerobic reductase with no homologs among the known aerobic/microaerobic reductases.

Thus, among 254 reference genomes, only 29 genomes (11%) are aerophilic, 111 (44%) are microaerophilic, and 115 (45%) are anaerobic. The details of the annotated respiratory reductases are captured as a subsystem “Respiration HGM” in the PubSEED database (Overbeek et al., 2005; Disz et al., 2010).

### Patterns of respiratory reductases in genomes

The reference genomes demonstrated perceptible variations in the distribution of respiratory reductases. The most common respiratory enzyme is the microaerobic reductase Cyd, which was found in 126 genomes (Table S1 in Supplementary Materials). Among anaerobic reductases, the fumarate reductase Frd was the most widespread and found in 93 of the studied genomes (36%). Other widespread respiratory reductases were nitrite reductase Nrf (found in 55 genomes, 22%), nitrate reductases Nar and Nap (found in 36 (15%) and 27 (11%) genomes, respectively), and DMSO/TMAO reductase Dms (found in 23 genomes, 9%). The other reductases were found in one to 19 of the studied genomes.

The largest number of respiratory reductases was found in *G. pamelaeae* and *Eggerthella* sp. 1_3_56FAA (57 and 27, respectively). These organisms are characterized by large copy numbers of reductases of the same type. Consequently, we identified 12 non-identical copies of Dms reductase in *G. pamelaeae*. Large numbers of reductases (between 10 and 20) were found in genomes of Enterobacteriaceae, Desulfovibrionaceae, and *Ralstonia* sp. 5_7_47FAA. In 151 genomes, one to seven reductases were found, and in 79 genomes, no reductases were detected. Two alternative explanations for this lack of respiratory reductases are possible: (1) these organisms contain only unknown reductases, which are not similar to any known reductases, or (2) these organisms exclusively use fermentation for energy production.

### Metabolism of nitrogen oxides: possible exchange pathways

**Figure 3:**
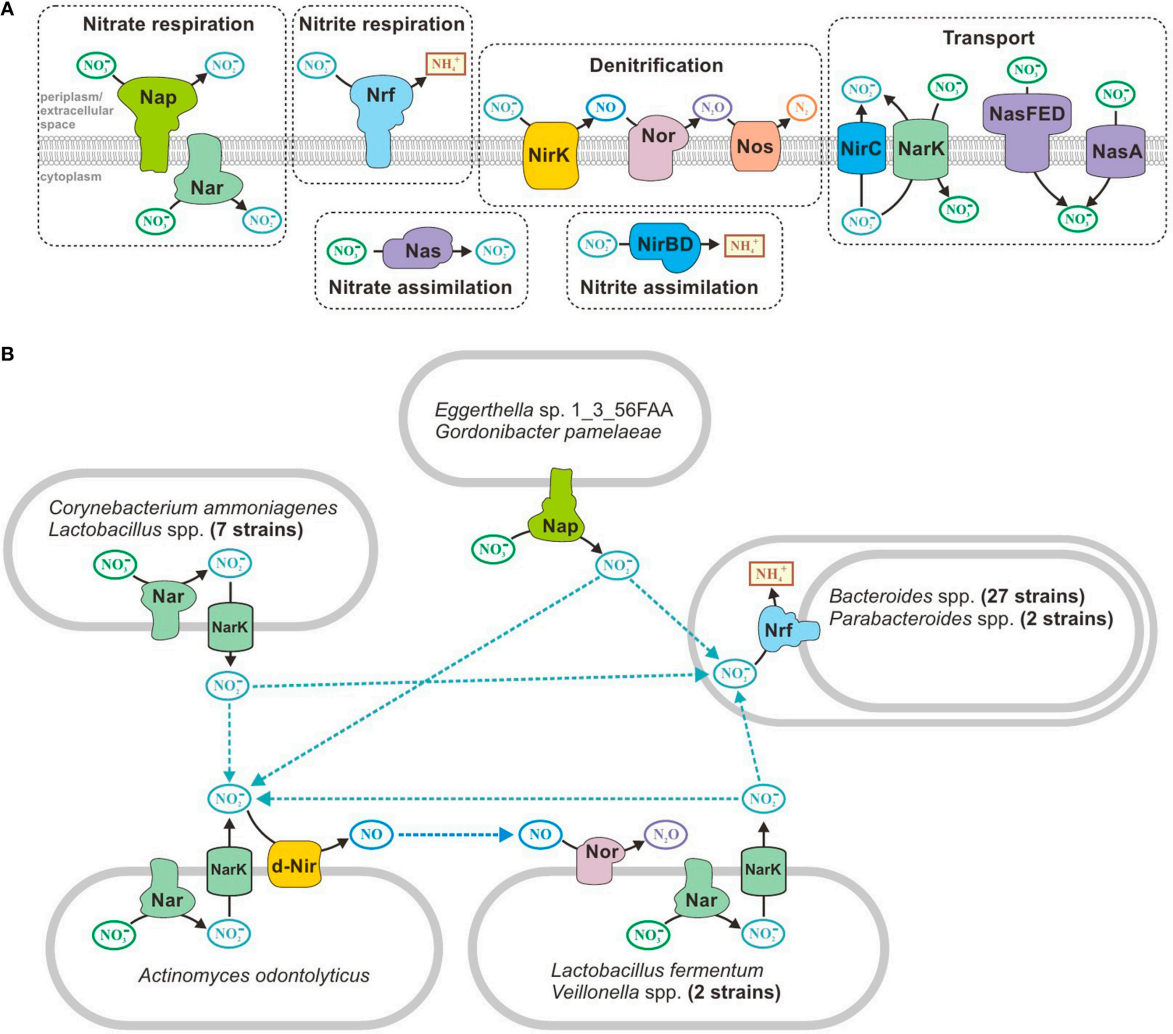
**Metabolism of nitrogen oxides in human gut microbes. (A)** All of the enzymes and transporters found in the reference genomes. **(B)** Predicted exchange pathways.

Various nitrogen oxides are often used as electron acceptors under anaerobic conditions (Figure 3A); therefore, microbes can use nitrate, nitrite, nitric oxide, and nitrous oxide (Ferguson, 1998; Gonzalez et al., 2006; Kraft et al., 2011; Luque-Almagro et al., 2011). During the analysis of anaerobic respiration in the human gut reference genomes, we found that certain studied genomes did not contain the full set of respiratory enzymes for the reduction of nitrate to ammonia or molecular nitrogen. For incomplete genomes, the lack of certain steps could be explained by the presence of corresponding genes in non-sequenced regions. However, for finished and most likely complete genomes, the explanation may include one of the following: the reduction of nitrogen oxides could be partially realized because of assimilatory enzymes, such as NasBA nitrate reductase (Ogawa et al., 1995) or NirBD nitrite reductase (Wang and Gunsalus, 2000); or microorganisms may be able to exchange byproducts of respiratory activity, such as one bacterial species reducing nitrate to nitrite and another species using the produced nitrite as an electron acceptor in a respiratory chain. To test this hypothesis, we analyzed all of the finished and most likely complete genomes for the presence of genes required for the reduction of nitrogen oxides, i.e., genes encoding the corresponding respiratory and assimilatory reductases and transport proteins.

Pathways for the reduction of nitrogen oxides were found in 73 finished and probably complete genomes (Table S5 in Supplementary Materials). Only 28 genomes contained all of the enzymes required for nitrate reduction to ammonia. The denitrifying pathway that reduces nitrate to molecular nitrogen was found exclusively in *Ralstonia* sp. 5_7_47FAA. Only nitrate (not nitrite) reductases were detected in 11 genomes. For example, in eight genomes of *Lactobacillus* spp., we found cytoplasmic nitrate reductase NarGHI and nitrite-nitrite antiporter NarK, which is required to nitrate/nitrite exchange between cytoplasm and periplasm. However, 29 genomes from the order Bacteroidales only included genes for periplasmic respiratory nitrite reductase Nrf. The *Actinomyces odontolyticus* genome also only contains genes for a partial denitrifying pathway, and this bacterium most likely can only reduce nitrate to nitric oxide. The most intriguing combination of reducing reactions was found in *Lactobacillus fermentum* IFO 3956 and *Veillonella* spp. These genomes have enzymes for the reduction of nitrate to nitrite and reduction of nitric oxide to nitrous oxide; however, they lack the enzymes for the intermediate stage in which nitrite is reduced to nitric oxide. Overall, 44 of the finished and probably complete genomes that were analyzed have partial pathways for the reduction of nitrogen oxides. All of these organisms can live under microaerobic conditions (see above) and are capable of inhabiting the same zone of the human intestine; therefore, no apparent barrier to metabolite exchange occurs between them. Thus, we propose that organisms with partial pathways could exchange nitrite and nitric oxide (Figure 3B).

### “Lego” principle for the organization of respiratory enzymes

Previous studies of respiratory reductases demonstrated a high degree of variability in the structure of reductase complexes. Thus, reductases of the same type can have different compositions of proteins in different genomes. The best known example of such variability is the periplasmic nitrite reductase Nrf, which only shares the catalytic subunit NrfA as a common component. In *E. coli*, this enzymatic complex consists of four subunits, NrfABCD (Page et al., 1990; Wang and Gunsalus, 2000) whereas in *Wolinella succinogenes* this complex consists of only two subunits, NrfAH (Einsle et al., 2000; Simon et al., 2000). Additionally, a large variability was observed for operons encoding respiratory reductases (for reference see refer to Table S2 in Supplementary Materials). Respiratory reductases are apparently organized similar to a Lego construction toy, where the final object is constructed from a set of standard building blocks.

In this work, we described cases that illustrated the variable structure of respiratory reductases in the reference genomes (Table S6 in Supplementary Materials). The predicted thiosulfate reductase Tsr is a perfect illustration of the “Lego” construction principle for respiratory reductases. The predicted catalytic subunit TsrA is a cytochrome *c-552/4*-like protein, and TsrHGIJ proteins are orthologs for NosDLFY proteins. In *Pseudomonas stutzeri*, these Nos proteins are components of the protein complex of nitrous oxide reductase. The protein TsrD is a homolog of NrfG, a protein involved in the maturation of the nitrite reductase complex in *E. coli* (Page et al., 1990; Wang and Gunsalus, 2000). TsrF belongs to the family of integral membrane proteins (Pfam ID: PF03916) that include the transmembrane subunits of nitrite (NrfD) and tetrathionate reductases (TtrC). TsrE is a representative of a large family of 4Fe-4S-cluster proteins (Pfam ID: PF13247). The members of this family are extremely widespread in redox complexes, including respiratory reductases for DMSO/TMAO (DmsB), nitrite (NrfC), and tetrathionate (TtrB). Thus, novel respiratory capacities can emerge by using components of the existing system, novel catalytic subunits and proteins from widespread protein families.

Furthermore, we identified certain unusual variations of the known respiratory reductases that included reductases lacking membrane components. For instance, in *G. pamelaeae* and *Eggerthella* sp. 1_3_56FAA, the *torA* gene was co-localized with the gene (named *torB*) for the iron-sulfur cluster protein, but membrane components were not found. Moreover, although identified TorA proteins (Table S2 in Supplementary Materials) were shown to be periplasmic, the TorAB complex was predicted to be cytoplasmic. In the same two genomes, the catalytic subunit for the predicted Ydh reductase, YdhV, was found alone without any membrane components. Similar to the previous case, YdhV was predicted to be cytoplasmic, which is in contrast to the predicted periplasmic subunit of the YdhYVWXUT complex of *E. coli*. The same situation was observed for the Dms system in *Clostridium asparagiforme* DSM 15981, *Clostridiales bacterium* 1_7_47FAA, and *B. bacterium* 1_1_47. In known Dms systems (Table S2 in Supplementary Materials), DmsAB proteins were periplasmic, whereas in subsequently identified genomes, no membrane components were found, and the DmsAB complex was predicted to be cytoplasmic.

The most intriguing situation was observed for the NrfA subunits. In the genomes of *G. pamelaeae* and *Eggerthella* sp. 1_3_56FAA, the predicted extracellular NrfAB proteins were found, whereas the predicted extracellular NrfA were detected in *Sutterella wadsworthensis* 3_1_45B and *B. bacterium* 1_1_47. In all cases, membrane components were not found. In 10 genomes of *Bacteroides* spp. and 2 genomes of *Parabacteroides* spp., the genes for the predicted cytoplasmic NrfA proteins were found in addition to genes for the membrane NrfA complex. A phylogenetic analysis (Figure S2 in Supplementary Materials) revealed that cytoplasmic copies of NrfA most likely appeared by duplication of the ancestor gene for the periplasmic membrane-bound NrfA. It is likely that the novel cytoplasmic copies are involved in nitrite assimilation and detoxification, which is analogous to the enterobacterial NirBD enzyme (Page et al., 1990).

The detection of respiratory reductases lacking membrane components raises a number of questions. For example, these reductases should have a source of electrons and should be able to interact with oxidases of electron donors or have oxidase domains. Nonetheless, all of the presumably soluble reductases found in this work had no additional domains compared with the cognate subunits from membrane complexes. However, additional domains are not always necessary, and a phylogenetic analysis of the PF00384 family (Figure 2) revealed examples that support this statement. Notably, these examples refer to experimentally analyzed proteins with known specificities and subcellular localization (Table S2 in Supplementary Materials). The NapA protein, which is a catalytic subunit of periplasmic nitrate reductase, is a close homolog of the soluble cytoplasmic assimilatory reductases NasA, NasC, and NarA, and its domain structure is conserved among all of these proteins. Another example is the soluble cytoplasmic protein BisC, which forms a branch with the catalytic subunits of periplasmic reductases specific for TMAO and DMSO/TMAO. Thus, detected non-membrane reductases may be soluble assimilatory reductases, and detected reductases without certain components can actually be cytoplasmic assimilatory reductases for the same electron acceptors as their membrane-bound respiratory versions.

We cannot conclude that these reductases actually lack membrane components. Co-localized genes were not found for membrane components, but we cannot be certain that they are absent from distant regions of the genome. The *nrfA* gene in bacteria from the family Shewanellaceae is in a separate location from the membrane component genes *nrfXBCD* (Rodionov et al., 2011). The soluble cytoplasmic complex DsrAB acts as the catalytic subunit of sulfite reductase, and the DsrMOJKP protein forms the membrane component of this reductase. The soluble and membrane components are encoded by separate and distant operons (Karkhoff-Schweizer et al., 1995), which is also observed for adenylyl sulfate reductase, where soluble and membrane components are formed by the AprAB and QmoABC proteins, respectively (Ramos et al., 2012).

The occurrence of predicted soluble reductases indicates several directions for further analysis. First, experimental analyses of these proteins would help to clarify their subcellular localization and substrate specificities. Second, more detailed genomic analyses might also provide additional information about these proteins. Various comparative genomics techniques, such as predicting regulatory interactions or analyzing gene co-occurrence in genomes, can help us to identify genes for membrane components that are located apart from the genes for catalytic subunits (reviewed in Osterman and Overbeek, 2003; Rodionov, 2007). In addition, the same comparative genomics approaches can corroborate the non-respiratory functions of these proteins, including their co-occurrence with genes for certain cytoplasmic pathways. For example, nitrite reductase without membrane components may be co-regulated with cytoplasmic genes for nitrogen assimilation but not with respiratory genes. Such co-regulation will support the assimilatory function of this enzyme. In addition, comparative genomics analyses can investigate related genomes from other habitats and do not have to focus on genomes for human gut microbiota.

## Discussion

The human gut microbiome has been intensively studied in recent years. Most of the studies have been based on the analysis of metagenome data, and they focus on the role of the gut microbiome in host nutrition, physiology, health, and disease. Additionally, the increased number of complete genome sequences for human gut microbes provides significant opportunities for computational analyses of these organisms and their interactions with the host organism. Surprisingly, no systematic analysis of respiratory genes in human gut microbes has been performed to date. However, bacterial respiration has been thoroughly studied in a number of model organisms (Richardson, 2000, 2013; Nealson et al., 2002), and some of these organisms, such as *E. coli*, are inhabitants of the human intestine. Additionally, substrates for the respiration of gut bacteria, such as TMAO (Wang et al., 2011) or tetrathionate (Winter et al., 2010a; Thiennimitr et al., 2011), were found to play crucial roles in human health and disease. Thus, the analysis of respiration in the human gut microbiome may also have practical significance.

In this work, we first systematically analyzed the respiratory reductases from a reference set of 254 genomic sequences for human gut microbes. The analysis of such a large number of individual genomes, which were completely or partially assembled, is a necessary link between two “traditional” approaches: analysis of model organisms and metagenome analysis. We analyzed the distribution of multiple aerobic, microaerobic, and anaerobic respiratory reductases as well as energy conserving hydrogenases that can act as reductases of electron acceptors under certain conditions. In addition to annotating known enzymes, we also predicted a novel microaerobic reductase and novel anaerobic thiosulfate reductase. Based on the distribution of various reductases, we divided the reference genomes into ecological niches. In addition, based on the genomic analysis, we reconstructed the network for the exchange of nitrogen oxides among various organisms that inhabit the human gut.

In combination with new results, this research inspired questions and yielded potential problems. The most obvious problem is related to the analyzed genomes and oxygen tolerance. The predicted capacity for oxygen use is consistent with the experimental data for only 85.8% of the analyzed genomes. When the predictions were compared with metadata extracted from the IMG database, the percentage of consistency was even less (76.1%). Surprisingly, no inconsistencies were observed when the predictions were compared with the IMG data derived from pathway assertion. Only certain cases of inconsistent results can be explained by the incompleteness of the analyzed genomes, and such inconsistencies will most likely be eliminated after the complete version of the genomes becomes available. Other inconsistencies, such as a lack of aerobic enzymes in the probably complete or finished genomes for organisms identified as aerobes, can be explained by the occurrence of unknown aerobic reductases. One such reductase was predicted in the *F. prausnitzii* genome in the present work. This reductase is non-homologous to any known aerobic or microaerobic reductase and could not be found by the normal comparative genomic procedures. Thus, to prevent controversies of this type, additional experimental data, including high-throughput data, should be available. In addition, an increased number of different techniques and approaches should be used during genome analysis, including genome content-based approaches and the use of metabolic network analysis (Manichaikul et al., 2009; Rolfsson et al., 2011, 2013; Heinken et al., 2014).

The bulk of inconsistencies included the following: a certain microorganism was considered to be an anaerobe according to the data derived from databases or the literature; however, the genomic analysis revealed the presence of genes for aerobic reductases. To prevent such inconsistency, a change of habit is required. First, dividing microbes into only two groups, aerobes and anaerobes, is too crude and usually based on fragmentary data. In most of the analyzed data from the literature (references are shown in Table S1 in Supplementary Materials), the microbe was assessed as an anaerobe (sometimes as a strong or obligate anaerobe) based only on its inability to grow in atmospheric oxygen concentrations. However, at least some of these organisms have been shown to grow in the presence of small concentrations of oxygen, and in these conditions, they use oxygen for energy production (Santos et al., 1993; Baughn and Malamy, 2004; Khan et al., 2012b). Second, metadata extracted from databases should be used with much greater skepticism. Although data based on genomic analyses cannot guarantee perfect accuracy, by using genome data, researchers can determine the primary source of such data (the genome sequence) and accept or reject the information with maximal rationality. Moreover, the amount of genomic data is rapidly increasing and provides a large area of activity. Third, maximal circumspection is required in the expansion of any statements to other organisms. However, although closely related organisms share a majority of biological characteristics, features such as oxygen use can be changed. For example, among the 20 finished genomes of *Lactobacillus* spp. analyzed in this research, only 16 shared genes for the microaerobic reductase Cyd (Table S1 in Supplementary Materials). Because all of these genomes are definitely complete and the *cyd* genes are clearly distinguishable, the absence of exclusive microaerobic reductase and changes in oxygen tolerance cannot be explained as artifacts of analysis. In the analysis and prediction of features as complex as lifestyle or ecological niche, the maximal number of different methods must be used, and a maximal level of criticism and attentiveness is demanded.

This study was focused on the analysis of a limited number of respiratory reductases in only 254 reference genomes. Such limitations are not an obstacle for determining a broader picture of respiratory capacities in human gut microbiomes, but they may require inferring certain details. For example, the patterns of respiratory reductases described above can be sufficiently changed after the addition of novel genomes to the reference set. Such possible skewing of results because of these limitations denotes two directions for further analysis. The first direction includes an addition of novel genomes of human gut microbes to the analysis and a re-analysis of draft genomes after their finished versions are complete. The second direction is an analysis of additional respiratory reductases. In this research, we concentrated on the analysis of known reductases and one novel microaerobic reductase, and anaerobic thiosulfate reductase Tsr was predicted occasionally and serendipitously. To improve our understanding of respiration in human gut microbes, systematic predictions of novel respiratory reductases should be performed. In this research, this type of analysis was performed for only one type of respiratory proteins, the catalytic molybdopterin-binding subunits of anaerobic reductases. The analysis of other families of respiratory proteins, such as reductase membrane components or heme and iron-sulfur cluster proteins, may be helpful for the prediction of novel respiratory reductases. The specificities of these novel reductases can be further predicted by various comparative genomics and genome-context based methods or identified by experimental analysis. In addition, the analysis of a family of related proteins can provide additional data for further investigations in evolutionary genomics.

The last direction recommended by the present work is to include data on respiration in the genomics-based metabolic models for individual microorganisms and the microbial community of the human intestine. However, before this inclusion, the complete respiratory chains should be reconstructed for a maximal number of genomes. Therefore, in the immediate future, this research will be extended to ATP synthases, respiratory dehydrogenases and their electron donors, and the genes for quinone biosynthesis. The final reconstruction of respiratory chains and their inclusion in metabolic models will further improve our knowledge and understanding of the metabolism of human gut bacteria as well as their interactions with each other and the human organism. We would also be extremely grateful if these predictions or others became a subject of experimental verification.

## Author contributions

Dmitry A. Ravcheev and Ines Thiele conceived and designed the research project and wrote the manuscript. Dmitry A. Ravcheev performed genomic analysis of the respiratory systems. All authors read and approved the final manuscript.

## Conflict of interest statement

The authors declare that the research was conducted in the absence of any commercial or financial relationships that could be construed as a potential conflict of interest.
