# Supplementary material for: Systematic genomic analysis reveals the complementary aerobic and anaerobic respiration capacities of the human gut microbiota
Source: Front Microbiol. 2014 Dec 5;5:674. doi: 10.3389/fmicb.2014.00674 (PMC4257093; doi:10.3389/fmicb.2014.00674)
Supplement: Supplementary file 2 [file Table2.DOCX]

**Table S2.** Previously known reductases, analyzed in this work. ^1^Reductases with the catalytic subunit belonging to molybdopterin oxidoreductase family (Pfam ID: PF00384).

| **Operon** | **Organism** | **Reference(s)** |
| --- | --- | --- |
| **Aerobic reductases** | | |
| ***1. Microaerobic cytochrome bd reductases*** | | |
| *cydAB* | *Escherichia coli* | ([Cotter et al., 1990](#_ENREF_19);[Iuchi et al., 1990](#_ENREF_38);[Bogachev et al., 1993](#_ENREF_9)) |
| *cydAB* | *Bacteroides fragilis* | ([Baughn and Malamy, 2004](#_ENREF_7)) |
| *cydAB* | *Streptomyces coelicolor* | ([Brekasis and Paget, 2003](#_ENREF_13)) |
| *cydAB* | *Allochromatium vinosum* | ([Dincturk et al., 2011](#_ENREF_24)) |
| *cydAB* | *Azotobacter vinelandii* | ([Kelly et al., 1990](#_ENREF_47)) |
| *cydAB* | *Bacillus subtilis* | ([Winstedt et al., 1998](#_ENREF_103);[Larsson et al., 2005](#_ENREF_53)) |
| *cydAB* | *Shewanella oneidensis* | ([Gralnick et al., 2005](#_ENREF_34)) |
| *cioAB* | *Pseudomonas aeruginosa* | ([Cooper et al., 2003](#_ENREF_18)) |
| *appCB* | *Escherichia coli* | ([Dassa et al., 1991](#_ENREF_23)) |
| *ythAB* | *Bacillus subtilis* | ([Winstedt et al., 1998](#_ENREF_103)) |
| ***2. Other aerobic reductases*** | |  |
| *cyoABCDE* | *Escherichia coli* | ([Abramson et al., 2000](#_ENREF_1)) |
| *cyaABCD* | *Acetobacter aceti* | ([Fukaya et al., 1993](#_ENREF_30)) |
| *qoxABCD* | *Bacillus subtilis* | ([Lemma et al., 1995](#_ENREF_54)) |
| *ctaCDEFG* | *Bacillus subtilis* | ([Sousa et al., 2013](#_ENREF_92)) |
| *ctaACD* | *Thermus thermophilus* | ([Mather et al., 1993](#_ENREF_62)) |
| *ctaD* | *Corynebacterium glutamicum* | ([Niebisch and Bott, 2001](#_ENREF_73)) |
| *coxBAEFGC* | *Bradyrhizobium japonicum* | ([Gabel and Maier, 1993](#_ENREF_31)) |
| *coxBAC* | *Nostoc* sp*.* PCC 7120 | ([Jones and Haselkorn, 2002](#_ENREF_42)) |
| *ctaCDE* | *Synechocystis* sp. PCC 6803 | ([Alge and Peschek, 1993](#_ENREF_3)) |
| *ccoNOQP* | *Pseudomonas aeruginosa* | ([Comolli and Donohue, 2004](#_ENREF_17)) |
| *ccoNOQP* | *Sinorhizobium meliloti* | ([Cabanes et al., 2000](#_ENREF_14)) |
| *ccoNOQP* | *Azorhizobium caulinodans* | ([Mandon et al., 1994](#_ENREF_60)) |
| *ccoNOQP* | *Bradyrhizobium japonicum* | ([Nellen-Anthamatten et al., 1998](#_ENREF_72);[Mesa et al., 2005](#_ENREF_65)) |
| *ccoNOQP* | *Rhizobium etli* | ([Lopez et al., 2001](#_ENREF_58)) |
| *ccoNOQP* | *Rhizobium leguminosarum* | ([Patschkowski et al., 1996](#_ENREF_77)) |
| *ccoNOQP* | *Paracoccus denitrificans* | ([Otten et al., 2001](#_ENREF_74)) |
| *ccoNOQP* | *Rhodobacter sphaeroides* | ([Toledo-Cuevas et al., 1998](#_ENREF_97);[Roh and Kaplan, 2002](#_ENREF_80)) |
| **Nitrate reductases** | | |
| *narGHJI* ^1^ | *Bacillus subtilis* | ([Cruz Ramos et al., 1995](#_ENREF_20);[Hoffmann et al., 1995](#_ENREF_37);[Nakano et al., 1996](#_ENREF_71)) |
| *narGHJI* ^1^ | *Staphylococcus aureus* | ([Dunman et al., 2001](#_ENREF_27)) |
| *narGHJI* ^1^ | *Pseudomonas stutzeri* | ([Vollack et al., 1999](#_ENREF_101)) |
| *narGHJI* ^1^ | *Escherichia coli* | ([Stewart, 1982](#_ENREF_93);[Li et al., 1994](#_ENREF_55);[Darwin et al., 1996](#_ENREF_22)) |
| *narZWYV* ^1^ | *Escherichia coli* | ([Bonnefoy and Demoss, 1994](#_ENREF_10);[Chang et al., 1999](#_ENREF_15)) |
| *narBCGHMJ* ^1^ | *Haloferax mediterranei* | ([Lledo et al., 2004](#_ENREF_56)) |
| *narCBGHM* ^1^ | *Haloarcula marismortui* | ([Yoshimatsu et al., 2002](#_ENREF_105)) |
| *narGHM* ^1^ | *Pyrobaculum aerophilum* | ([Afshar et al., 2001](#_ENREF_2)) |
| *narGHM* ^1^ | *Archaeoglobus fulgidus* | ([Dridge et al., 2006](#_ENREF_26)) |
| *napFDAGHBC* ^1^ | *Escherichia coli* | ([Stewart et al., 2002](#_ENREF_95);[Stewart and Bledsoe, 2005](#_ENREF_94)) |
| *napEDABC* ^1^ | *Bradyrhizobium japonicum* | ([Robles et al., 2006](#_ENREF_79)) |
| *napCBADE* ^1^ | *Cupriavidus necator* | ([Siddiqui et al., 1993](#_ENREF_85)) |
| *napAGHD* ^1^ | *Wolinella succinogenes* | ([Simon et al., 2003](#_ENREF_91)) |
| *napCMADGH* ^1^ | *Desulfovibrio desulfuricans* | ([Marietou et al., 2005](#_ENREF_61)) |

| **Operon** | **Organism** | **Reference(s)** |
| --- | --- | --- |
| **Nitrite reductases** | | |
| *nrfABCDEFG* | *Escherichia coli* | ([Page et al., 1990](#_ENREF_75);[Wang and Gunsalus, 2000](#_ENREF_102)) |
| *nrfHAIE* | *Wolinella succinogenes* | ([Einsle et al., 2000](#_ENREF_28);[Simon et al., 2000](#_ENREF_89)) |
| *nirK* | *Bradyrhizobium japonicum* | ([Velasco et al., 2001](#_ENREF_100)) |
| *nirSTNM* | *Pseudomonas stutzeri* | ([Jungst et al., 1991](#_ENREF_44)) |
| **Nitric oxide reductase** | | |
| *norCBQD* | *Pseudomonas aeruginosa* | ([Arai et al., 1995](#_ENREF_5)) |
| **Nitrous oxide reductase** | | |
| *nosRZDFYL* | *Pseudomonas stutzeri* | ([Zumft et al., 1990](#_ENREF_107)) |
| **Tetrationate reductase** | | |
| *ttrBCA* ^1^ | *Salmonella typhimurium* | ([Hensel et al., 1999](#_ENREF_36)) |
| **Thiosulfate reductase** | | |
| *phsABC* ^1^ | *Salmonella typhimurium* | ([Clark and Barrett, 1987](#_ENREF_16)) |
| **Polysulfite reductase** | | |
| *psrABC* ^1^ | *Wolinella succinogenes* | ([Krafft et al., 1992](#_ENREF_49)) |
| **Sulfite reductase** | | |
| *dsrAB, dsrMKJOP* | *Archaeoglobus fulgidus; Desulfovibrio vulgaris* | ([Karkhoff-Schweizer et al., 1995](#_ENREF_46)) |
| **Adenylyl sulfate reductase** | | |
| *aprAB, qmoABC* | *Desulfovibrio desulfuricans* | ([Ramos et al., 2012](#_ENREF_78)) |
| **Heterodisulfide reductases** | | |
| *hdrABC* | *Methanosarcina barkeri* | ([Kunkel et al., 1997](#_ENREF_50)) |
| *hdrFE* | *Methanosarcina barkeri* | ([Kunkel et al., 1997](#_ENREF_50)) |
| **Dimethyl sulfoxide / Trimethylamine N-oxide reductases** | | |
| *dmsEFABGH* ^1^ | *Shewanella oneidensis* | ([Gralnick et al., 2005](#_ENREF_34)) |
| *dmsABCDF* ^1^ | *Haemophilus influenzae* | ([Loosmore et al., 1996](#_ENREF_57)) |
| *dmsABC* ^1^ | *Escherichia coli* | ([Sambasivarao et al., 1990](#_ENREF_82);[Trieber et al., 1994](#_ENREF_98);[Tseng et al., 1996](#_ENREF_99)) |
| *dmsEABCD* ^1^ | *Halobacterium* sp. NRC-1 | ([Muller and DasSarma, 2005](#_ENREF_70)) |
| *ddhABDC* ^1^ | *Rhodovulum sulfidophilum* | ([McDevitt et al., 2002](#_ENREF_63)) |
| *dorABC* ^1^ | *Rhodobacter sphaeroides* | ([Mouncey et al., 1997](#_ENREF_66);[Mouncey and Kaplan, 1998b](#_ENREF_68);[a](#_ENREF_67);[Yamamoto et al., 2001](#_ENREF_104)) |
| **Trimethylamine N-oxide reductases** | | |
| *torYZ* ^1^ | *Escherichia coli* | ([Gon et al., 2000](#_ENREF_33)) |
| *torCAD* ^1^ | *Escherichia coli* | ([Iuchi and Lin, 1987](#_ENREF_39);[Silvestro et al., 1989](#_ENREF_87);[Mejean et al., 1994](#_ENREF_64);[Simon et al., 1995](#_ENREF_88);[Jourlin et al., 1996](#_ENREF_43);[Santini et al., 1998](#_ENREF_83)) |
| *torECAD* ^1^ | *Shewanella oneidensis* | ([Czjzek et al., 1998](#_ENREF_21);[Dos Santos et al., 1998](#_ENREF_25);[Bordi et al., 2003](#_ENREF_12);[Bordi et al., 2004](#_ENREF_11)) |
| **Fumarate reductase** | | |
| *frdABCD* | *Escherichia coli* | ([Jones and Gunsalus, 1987](#_ENREF_41);[Kalman and Gunsalus, 1989](#_ENREF_45)) |
| *frdCAB* | *Bacteroides fragilis* | ([Baughn and Malamy, 2003](#_ENREF_6)) |
| *frdCAB* | *Wolinella succinogenes* | ([Kortner et al., 1990](#_ENREF_48);[Simon et al., 1998](#_ENREF_90);[Lancaster, 2001](#_ENREF_52)) |
| *frdCAB* | *Helicobacter pylori* | ([Ge et al., 1997](#_ENREF_32)) |
| **H_2_-evolving hydrogenases** | | |
| *hycBCDEFG* | *Escherichia coli* | ([Maeda et al., 2007](#_ENREF_59)) |
| *hyfABCDEFGHI* | *Escherichia coli* | ([Andrews et al., 1997](#_ENREF_4)) |
| *mbhABCDEFGHIJKLMN* | *Pyrococcus furiosus* | ([Silva et al., 2000](#_ENREF_86)) |
| *echABCDEF* | *Methanosarcina barkeri* | ([Kunkel et al., 1998](#_ENREF_51)) |
| *cooMKLXUH* | *Rhodospirillum rubrum* | ([Fox et al., 1996](#_ENREF_29)) |
| **Selenate reductases** | | |
| *ynfEFGH-dmsD* ^1^ | *Salmonella typhimurium* | ([Guymer et al., 2009](#_ENREF_35)) |
| *serABCD* ^1^ | *Thauera selenatis* | ([Schroder et al., 1997](#_ENREF_84)) |
| **Operon** | **Organism** | **Reference(s)** |
| **Arsenate reductases** | | |
| *arrAB* ^1^ | *Shewanella* sp. ANA-3 | ([Saltikov and Newman, 2003](#_ENREF_81)) |
| *arxB_2_ABC* ^1^ | *Ectothiorhodospira* sp. PHS-1 | ([Zargar et al., 2012](#_ENREF_106)) |
| *aioAB* ^1^ | *Alcaligenes faecalis* | ([Muller et al., 2003](#_ENREF_69)) |
| **Ethylbenzene reductase** | | |
| *ebdABCD* ^1^ | *Azoarcus sp.* EbN1 | ([Johnson et al., 2001](#_ENREF_40)) |
| **Chlorate reductase** | | |
| *clrADBC* ^1^ | *Ideonella dechloratans* | ([Thorell et al., 2003](#_ENREF_96)) |
| **Perchlorate reductase** | | |
| *pcrABCD* ^1^ | *Dechloromonas aromatica* | ([Bender et al., 2005](#_ENREF_8)) |
| **Predicted reductase (unknown specificity)** | | |
| *ydhYVWXUT* | *Escherichia coli* | ([Partridge et al., 2008](#_ENREF_76)) |

# References

Abramson, J., Riistama, S., Larsson, G., Jasaitis, A., Svensson-Ek, M., Laakkonen, L., Puustinen, A., Iwata, S., and Wikstrom, M. (2000). The structure of the ubiquinol oxidase from *Escherichia coli* and its ubiquinone binding site. *Nat Struct Biol* 7**,** 910-917.

Afshar, S., Johnson, E., De Vries, S., and Schroder, I. (2001). Properties of a thermostable nitrate reductase from the hyperthermophilic archaeon *Pyrobaculum aerophilum*. *J Bacteriol* 183**,** 5491-5495.

Alge, D., and Peschek, G.A. (1993). Characterization of a *cta/CDE* operon-like genomic region encoding subunits I-III of the cytochrome c oxidase of the cyanobacterium Synechocystis PCC 6803. *Biochem Mol Biol Int* 29**,** 511-525.

Andrews, S.C., Berks, B.C., Mcclay, J., Ambler, A., Quail, M.A., Golby, P., and Guest, J.R. (1997). A 12-cistron *Escherichia coli operon* (*hyf*) encoding a putative proton-translocating formate hydrogenlyase system. *Microbiology* 143 ( Pt 11)**,** 3633-3647.

Arai, H., Igarashi, Y., and Kodama, T. (1995). The structural genes for nitric oxide reductase from *Pseudomonas aeruginosa*. *Biochim Biophys Acta* 1261**,** 279-284.

Baughn, A.D., and Malamy, M.H. (2003). The essential role of fumarate reductase in haem-dependent growth stimulation of *Bacteroides fragilis*. *Microbiology* 149**,** 1551-1558.

Baughn, A.D., and Malamy, M.H. (2004). The strict anaerobe *Bacteroides fragilis* grows in and benefits from nanomolar concentrations of oxygen. *Nature* 427**,** 441-444.

Bender, K.S., Shang, C., Chakraborty, R., Belchik, S.M., Coates, J.D., and Achenbach, L.A. (2005). Identification, characterization, and classification of genes encoding perchlorate reductase. *J Bacteriol* 187**,** 5090-5096.

Bogachev, A.V., Murtazina, R.A., and Skulachev, V.P. (1993). Cytochrome *d* induction in *Escherichia coli* growing under unfavorable conditions. *FEBS Letters* 336**,** 75-78.

Bonnefoy, V., and Demoss, J.A. (1994). Nitrate reductases in *Escherichia coli*. *Antonie Van Leeuwenhoek* 66**,** 47-56.

Bordi, C., Ansaldi, M., Gon, S., Jourlin-Castelli, C., Iobbi-Nivol, C., and Mejean, V. (2004). Genes regulated by TorR, the trimethylamine oxide response regulator of *Shewanella oneidensis*. *J Bacteriol* 186**,** 4502-4509.

Bordi, C., Iobbi-Nivol, C., Mejean, V., and Patte, J.C. (2003). Effects of ISSo2 insertions in structural and regulatory genes of the trimethylamine oxide reductase of *Shewanella oneidensis*. *J Bacteriol* 185**,** 2042-2045.

Brekasis, D., and Paget, M.S. (2003). A novel sensor of NADH/NAD^+^ redox poise in *Streptomyces coelicolor* A3(2). *EMBO Journal* 22**,** 4856-4865.

Cabanes, D., Boistard, P., and Batut, J. (2000). Identification of *Sinorhizobium meliloti* genes regulated during symbiosis. *J Bacteriol* 182**,** 3632-3637.

Chang, L., Wei, L.I., Audia, J.P., Morton, R.A., and Schellhorn, H.E. (1999). Expression of the *Escherichia coli* NRZ nitrate reductase is highly growth phase dependent and is controlled by RpoS, the alternative vegetative sigma factor. *Mol Microbiol* 34**,** 756-766.

Clark, M.A., and Barrett, E.L. (1987). The *phs* gene and hydrogen sulfide production by *Salmonella typhimurium*. *Journal of Bacteriology* 169**,** 2391-2397.

Comolli, J.C., and Donohue, T.J. (2004). Differences in two *Pseudomonas aeruginosa* cbb3 cytochrome oxidases. *Mol Microbiol* 51**,** 1193-1203.

Cooper, M., Tavankar, G.R., and Williams, H.D. (2003). Regulation of expression of the cyanide-insensitive terminal oxidase in *Pseudomonas aeruginosa*. *Microbiology* 149**,** 1275-1284.

Cotter, P.A., Chepuri, V., Gennis, R.B., and Gunsalus, R.P. (1990). Cytochrome o (*cyoABCDE*) and d (*cydAB*) oxidase gene expression in *Escherichia coli* is regulated by oxygen, pH, and the *fnr* gene product. *Journal of Bacteriology* 172**,** 6333-6338.

Cruz Ramos, H., Boursier, L., Moszer, I., Kunst, F., Danchin, A., and Glaser, P. (1995). Anaerobic transcription activation in *Bacillus subtilis*: identification of distinct FNR-dependent and -independent regulatory mechanisms. *EMBO J* 14**,** 5984-5994.

Czjzek, M., Dos Santos, J.P., Pommier, J., Giordano, G., Mejean, V., and Haser, R. (1998). Crystal structure of oxidized trimethylamine N-oxide reductase from *Shewanella massilia* at 2.5 A resolution. *J Mol Biol* 284**,** 435-447.

Darwin, A.J., Li, J., and Stewart, V. (1996). Analysis of nitrate regulatory protein NarL-binding sites in the *fdnG* and *narG* operon control regions of *Escherichia coli* K-12. *Molecular Microbiology* 20**,** 621-632.

Dassa, J., Fsihi, H., Marck, C., Dion, M., Kieffer-Bontemps, M., and Boquet, P.L. (1991). A new oxygen-regulated operon in *Escherichia coli* comprises the genes for a putative third cytochrome oxidase and for pH 2.5 acid phosphatase (*appA*). *Mol Gen Genet* 229**,** 341-352.

Dincturk, H.B., Demir, V., and Aykanat, T. (2011). Bd oxidase homologue of photosynthetic purple sulfur bacterium *Allochromatium vinosum* is co-transcribed with a nitrogen fixation related gene. *Antonie Van Leeuwenhoek* 99**,** 211-220.

Dos Santos, J.P., Iobbi-Nivol, C., Couillault, C., Giordano, G., and Mejean, V. (1998). Molecular analysis of the trimethylamine N-oxide (TMAO) reductase respiratory system from a *Shewanella* species. *J Mol Biol* 284**,** 421-433.

Dridge, E.J., Richardson, D.J., Lewis, R.J., and Butler, C.S. (2006). Developing structure-based models to predict substrate specificity of D-group (Type II) molybdenum enzymes: application to a molybdo-enzyme of unknown function from *Archaeoglobus fulgidus*. *Biochem Soc Trans* 34**,** 118-121.

Dunman, P.M., Murphy, E., Haney, S., Palacios, D., Tucker-Kellogg, G., Wu, S., Brown, E.L., Zagursky, R.J., Shlaes, D., and Projan, S.J. (2001). Transcription profiling-based identification of *Staphylococcus aureus* genes regulated by the *agr* and/or *sarA* loci. *J Bacteriol* 183**,** 7341-7353.

Einsle, O., Stach, P., Messerschmidt, A., Simon, J., Kroger, A., Huber, R., and Kroneck, P.M. (2000). Cytochrome c nitrite reductase from *Wolinella succinogenes*. Structure at 1.6 A resolution, inhibitor binding, and heme-packing motifs. *J Biol Chem* 275**,** 39608-39616.

Fox, J.D., Kerby, R.L., Roberts, G.P., and Ludden, P.W. (1996). Characterization of the CO-induced, CO-tolerant hydrogenase from *Rhodospirillum rubrum* and the gene encoding the large subunit of the enzyme. *J Bacteriol* 178**,** 1515-1524.

Fukaya, M., Tayama, K., Tamaki, T., Ebisuya, H., Okumura, H., Kawamura, Y., Horinouchi, S., and Beppu, T. (1993). Characterization of a cytochrome a1 that functions as a ubiquinol oxidase in *Acetobacter aceti*. *J Bacteriol* 175**,** 4307-4314.

Gabel, C., and Maier, R.J. (1993). Oxygen-dependent transcriptional regulation of cytochrome aa3 in *Bradyrhizobium japonicum*. *J Bacteriol* 175**,** 128-132.

Ge, Z., Jiang, Q., Kalisiak, M.S., and Taylor, D.E. (1997). Cloning and functional characterization of *Helicobacter pylori* fumarate reductase operon comprising three structural genes coding for subunits C, A and B. *Gene* 204**,** 227-234.

Gon, S., Patte, J.C., Mejean, V., and Iobbi-Nivol, C. (2000). The *torYZ* (*yecK-bisZ*) operon encodes a third respiratory trimethylamine N-oxide reductase in *Escherichia coli*. *Journal of Bacteriology* 182**,** 5779-5786.

Gralnick, J.A., T., B.C., and Newman, D.K. (2005). Anaerobic regulation by an atypical Arc system in *Shewanella oneidensis*. *Molecular Microbiology* 56**,** 1347-1357.

Guymer, D., Maillard, J., and Sargent, F. (2009). A genetic analysis of in vivo selenate reduction by *Salmonella enterica* serovar *Typhimurium* LT2 and *Escherichia coli* K12. *Archives of Microbiology* 191**,** 519-528.

Hensel, M., Hinsley, A.P., Nikolaus, T., Sawers, G., and Berks, B.C. (1999). The genetic basis of tetrathionate respiration in *Salmonella typhimurium*. *Mol Microbiol* 32**,** 275-287.

Hoffmann, T., Troup, B., Szabo, A., Hungerer, C., and Jahn, D. (1995). The anaerobic life of *Bacillus subtilis*: cloning of the genes encoding the respiratory nitrate reductase system. *FEMS Microbiol Lett* 131**,** 219-225.

Iuchi, S., Chepuri, V., Fu, H.A., Gennis, R.B., and Lin, E.C. (1990). Requirement for terminal cytochromes in generation of the aerobic signal for the arc regulatory system in *Escherichia coli*: study utilizing deletions and lac fusions of *cyo* and *cyd*. *Journal of Bacteriology* 172**,** 6020-6025.

Iuchi, S., and Lin, E.C. (1987). The *narL* gene product activates the nitrate reductase operon and represses the fumarate reductase and trimethylamine N-oxide reductase operons in *Escherichia coli*. *Proceedings of the National Academy of Sciences of the United States of America* 84**,** 3901-3905.

Johnson, H.A., Pelletier, D.A., and Spormann, A.M. (2001). Isolation and characterization of anaerobic ethylbenzene dehydrogenase, a novel Mo-Fe-S enzyme. *J Bacteriol* 183**,** 4536-4542.

Jones, H.M., and Gunsalus, R.P. (1987). Regulation of *Escherichia coli* fumarate reductase (*frdABCD*) operon expression by respiratory electron acceptors and the *fnr* gene product. *Journal of Bacteriology* 169**,** 3340-3349.

Jones, K.M., and Haselkorn, R. (2002). Newly identified cytochrome c oxidase operon in the nitrogen-fixing cyanobacterium *Anabaena* sp. strain PCC 7120 specifically induced in heterocysts. *J Bacteriol* 184**,** 2491-2499.

Jourlin, C., Simon, G., Pommier, J., Chippaux, M., and Mejean, V. (1996). The periplasmic TorT protein is required for trimethylamine N-oxide reductase gene induction in *Escherichia coli*. *J Bacteriol* 178**,** 1219-1223.

Jungst, A., Wakabayashi, S., Matsubara, H., and Zumft, W.G. (1991). The *nirSTBM* region coding for cytochrome cd1-dependent nitrite respiration of *Pseudomonas stutzeri* consists of a cluster of mono-, di-, and tetraheme proteins. *FEBS Lett* 279**,** 205-209.

Kalman, L.V., and Gunsalus, R.P. (1989). Identification of a second gene involved in global regulation of fumarate reductase and other nitrate-controlled genes for anaerobic respiration in *Escherichia coli*. *Journal of Bacteriology* 171**,** 3810.

Karkhoff-Schweizer, R.R., Huber, D.P., and Voordouw, G. (1995). Conservation of the genes for dissimilatory sulfite reductase from *Desulfovibrio vulgaris* and *Archaeoglobus fulgidus* allows their detection by PCR. *Appl Environ Microbiol* 61**,** 290-296.

Kelly, M.J., Poole, R.K., Yates, M.G., and Kennedy, C. (1990). Cloning and mutagenesis of genes encoding the cytochrome bd terminal oxidase complex in *Azotobacter vinelandii*: mutants deficient in the cytochrome d complex are unable to fix nitrogen in air. *Journal of Bacteriology* 172**,** 6010-6019.

Kortner, C., Lauterbach, F., Tripier, D., Unden, G., and Kroger, A. (1990). *Wolinella succinogenes* fumarate reductase contains a dihaem cytochrome b. *Mol Microbiol* 4**,** 855-860.

Krafft, T., Bokranz, M., Klimmek, O., Schroder, I., Fahrenholz, F., Kojro, E., and Kroger, A. (1992). Cloning and nucleotide sequence of the *psrA* gene of *Wolinella succinogene*s polysulphide reductase. *Eur J Biochem* 206**,** 503-510.

Kunkel, A., Vaupel, M., Heim, S., Thauer, R.K., and Hedderich, R. (1997). Heterodisulfide reductase from methanol-grown cells of *Methanosarcina barkeri* is not a flavoenzyme. *Eur J Biochem* 244**,** 226-234.

Kunkel, A., Vorholt, J.A., Thauer, R.K., and Hedderich, R. (1998). An *Escherichia coli* hydrogenase-3-type hydrogenase in methanogenic archaea. *Eur J Biochem* 252**,** 467-476.

Lancaster, C.R. (2001). Succinate:quinone oxidoreductases--what can we learn from Wolinella succinogenes quinol:fumarate reductase? *FEBS Lett* 504**,** 133-141.

Larsson, J.T., Rogstam, A., and Von Wachenfeldt, C. (2005). Coordinated patterns of cytochrome bd and lactate dehydrogenase expression in *Bacillus subtilis*. *Microbiology* 151**,** 3323-3335.

Lemma, E., Simon, J., Schagger, H., and Kroger, A. (1995). Properties of the menaquinol oxidase (Qox) and of qox deletion mutants of *Bacillus subtilis*. *Archives of Microbiology* 163**,** 432-438.

Li, J., Kustu, S., and Stewart, V. (1994). In vitro interaction of nitrate-responsive regulatory protein NarL with DNA target sequences in the *fdnG*, *narG*, *narK* and *frdA* operon control regions of *Escherichia coli* K-12. *Journal of Molecular Biology* 241**,** 150-165.

Lledo, B., Martinez-Espinosa, R.M., Marhuenda-Egea, F.C., and Bonete, M.J. (2004). Respiratory nitrate reductase from haloarchaeon *Haloferax mediterranei*: biochemical and genetic analysis. *Biochim Biophys Acta* 1674**,** 50-59.

Loosmore, S.M., Shortreed, J.M., Coleman, D.C., England, D.M., and Klein, M.H. (1996). Sequences of the genes encoding the A, B and C subunits of the *Haemophilus influenzae* dimethylsulfoxide reductase complex. *Gene* 169**,** 137-138.

Lopez, O., Morera, C., Miranda-Rios, J., Girard, L., Romero, D., and Soberon, M. (2001). Regulation of gene expression in response to oxygen in *Rhizobium etli*: role of FnrN in *fixNOQP* expression and in symbiotic nitrogen fixation. *Journal of Bacteriology* 183**,** 6999-7006.

Maeda, T., Sanchez-Torres, V., and Wood, T.K. (2007). Escherichia coli hydrogenase 3 is a reversible enzyme possessing hydrogen uptake and synthesis activities. *Appl Microbiol Biotechnol* 76**,** 1035-1042.

Mandon, K., Kaminski, P.A., and Elmerich, C. (1994). Functional analysis of the *fixNOQP* region of *Azorhizobium caulinodans*. *J Bacteriol* 176**,** 2560-2568.

Marietou, A., Richardson, D., Cole, J., and Mohan, S. (2005). Nitrate reduction by *Desulfovibrio desulfuricans*: a periplasmic nitrate reductase system that lacks NapB, but includes a unique tetraheme c-type cytochrome, NapM. *FEMS Microbiol Lett* 248**,** 217-225.

Mather, M.W., Springer, P., Hensel, S., Buse, G., and Fee, J.A. (1993). Cytochrome oxidase genes from *Thermus thermophilus*. Nucleotide sequence of the fused gene and analysis of the deduced primary structures for subunits I and III of cytochrome caa3. *J Biol Chem* 268**,** 5395-5408.

Mcdevitt, C.A., Hugenholtz, P., Hanson, G.R., and Mcewan, A.G. (2002). Molecular analysis of dimethyl sulphide dehydrogenase from *Rhodovulum sulfidophilum*: its place in the dimethyl sulphoxide reductase family of microbial molybdopterin-containing enzymes. *Mol Microbiol* 44**,** 1575-1587.

Mejean, V., Iobbi-Nivol, C., Lepelletier, M., Giordano, G., Chippaux, M., and Pascal, M.C. (1994). TMAO anaerobic respiration in *Escherichia coli*: involvement of the *tor* operon. *Molecular Microbiology* 11**,** 1169-1179.

Mesa, S., Ucurum, Z., Hennecke, H., and Fischer, H.M. (2005). Transcription activation in vitro by the *Bradyrhizobium japonicum* regulatory protein FixK2. *J Bacteriol* 187**,** 3329-3338.

Mouncey, N.J., Choudhary, M., and Kaplan, S. (1997). Characterization of genes encoding dimethyl sulfoxide reductase of *Rhodobacter sphaeroides* 2.4.1T: an essential metabolic gene function encoded on chromosome II. *J Bacteriol* 179**,** 7617-7624.

Mouncey, N.J., and Kaplan, S. (1998a). Cascade regulation of dimethyl sulfoxide reductase (*dor*) gene expression in the facultative phototroph *Rhodobacter sphaeroides* 2.4.1T. *J Bacteriol* 180**,** 2924-2930.

Mouncey, N.J., and Kaplan, S. (1998b). Redox-dependent gene regulation in *Rhodobacter sphaeroides* 2.4.1(T): effects on dimethyl sulfoxide reductase (*dor*) gene expression. *J Bacteriol* 180**,** 5612-5618.

Muller, D., Lievremont, D., Simeonova, D.D., Hubert, J.C., and Lett, M.C. (2003). Arsenite oxidase aox genes from a metal-resistant beta-proteobacterium. *J Bacteriol* 185**,** 135-141.

Muller, J.A., and Dassarma, S. (2005). Genomic analysis of anaerobic respiration in the archaeon *Halobacterium* sp. strain NRC-1: dimethyl sulfoxide and trimethylamine N-oxide as terminal electron acceptors. *J Bacteriol* 187**,** 1659-1667.

Nakano, M.M., Zuber, P., Glaser, P., Danchin, A., and Hulett, F.M. (1996). Two-component regulatory proteins ResD-ResE are required for transcriptional activation of fnr upon oxygen limitation in *Bacillus subtilis*. *J Bacteriol* 178**,** 3796-3802.

Nellen-Anthamatten, D., Rossi, P., Preisig, O., Kullik, I., Babst, M., Fischer, H.M., and Hennecke, H. (1998). *Bradyrhizobium japonicum* FixK2, a crucial distributor in the FixLJ-dependent regulatory cascade for control of genes inducible by low oxygen levels. *J Bacteriol* 180**,** 5251-5255.

Niebisch, A., and Bott, M. (2001). Molecular analysis of the cytochrome *bc1-aa3* branch of the *Corynebacterium glutamicum* respiratory chain containing an unusual diheme cytochrome *c1*. *Arch Microbiol* 175**,** 282-294.

Otten, M.F., Stork, D.M., Reijnders, W.N., Westerhoff, H.V., and Van Spanning, R.J. (2001). Regulation of expression of terminal oxidases in *Paracoccus denitrificans*. *Eur J Biochem* 268**,** 2486-2497.

Page, L., Griffiths, L., and Cole, J.A. (1990). Different physiological roles of two independent pathways for nitrite reduction to ammonia by enteric bacteria. *Archives of Microbiology* 154**,** 349-354.

Partridge, J.D., Browning, D.F., Xu, M., Newnham, L.J., Scott, C., Roberts, R.E., Poole, R.K., and Green, J. (2008). Characterization of the *Escherichia coli* K-12 *ydhYVWXUT* operon: regulation by FNR, NarL and NarP. *Microbiology* 154**,** 608-618.

Patschkowski, T., Schluter, A., and Priefer, U.B. (1996). *Rhizobium leguminosarum* bv. viciae contains a second fnr/fixK-like gene and an unusual fixL homologue. *Mol Microbiol* 21**,** 267-280.

Ramos, A.R., Keller, K.L., Wall, J.D., and Pereira, I.A. (2012). The Membrane QmoABC Complex Interacts Directly with the Dissimilatory Adenosine 5'-Phosphosulfate Reductase in Sulfate Reducing Bacteria. *Front Microbiol* 3**,** 137.

Robles, E.F., Sanchez, C., Bonnard, N., Delgado, M.J., and Bedmar, E.J. (2006). The *Bradyrhizobium japonicum napEDABC* genes are controlled by the FixLJ-FixK(2)-NnrR regulatory cascade. *Biochemical Society Transactions* 34**,** 108-110.

Roh, J.H., and Kaplan, S. (2002). Interdependent expression of the *ccoNOQP-rdxBHIS* loci in *Rhodobacter sphaeroides* 2.4.1. *J Bacteriol* 184**,** 5330-5338.

Saltikov, C.W., and Newman, D.K. (2003). Genetic identification of a respiratory arsenate reductase. *Proc Natl Acad Sci U S A* 100**,** 10983-10988.

Sambasivarao, D., Scraba, D.G., Trieber, C., and Weiner, J.H. (1990). Organization of dimethyl sulfoxide reductase in the plasma membrane of *Escherichia coli*. *J Bacteriol* 172**,** 5938-5948.

Santini, C.L., Ize, B., Chanal, A., Muller, M., Giordano, G., and Wu, L.F. (1998). A novel sec-independent periplasmic protein translocation pathway in *Escherichia coli*. *EMBO J* 17**,** 101-112.

Schroder, I., Rech, S., Krafft, T., and Macy, J.M. (1997). Purification and characterization of the selenate reductase from *Thauera selenatis*. *J Biol Chem* 272**,** 23765-23768.

Siddiqui, R.A., Warnecke-Eberz, U., Hengsberger, A., Schneider, B., Kostka, S., and Friedrich, B. (1993). Structure and function of a periplasmic nitrate reductase in *Alcaligenes eutrophus* H16. *J Bacteriol* 175**,** 5867-5876.

Silva, P.J., Van Den Ban, E.C., Wassink, H., Haaker, H., De Castro, B., Robb, F.T., and Hagen, W.R. (2000). Enzymes of hydrogen metabolism in *Pyrococcus furiosus*. *Eur J Biochem* 267**,** 6541-6551.

Silvestro, A., Pommier, J., Pascal, M.C., and Giordano, G. (1989). The inducible trimethylamine N-oxide reductase of *Escherichia coli* K12: its localization and inducers. *Biochim Biophys Acta* 999**,** 208-216.

Simon, G., Jourlin, C., Ansaldi, M., Pascal, M.C., Chippaux, M., and Mejean, V. (1995). Binding of the TorR regulator to *cis*-acting direct repeats activates *tor* operon expression. *Molecular Microbiology* 17**,** 971-980.

Simon, J., Gross, R., Einsle, O., Kroneck, P.M., Kroger, A., and Klimmek, O. (2000). A NapC/NirT-type cytochrome c (NrfH) is the mediator between the quinone pool and the cytochrome c nitrite reductase of *Wolinella succinogenes*. *Mol Microbiol* 35**,** 686-696.

Simon, J., Gross, R., Ringel, M., Schmidt, E., and Kroger, A. (1998). Deletion and site-directed mutagenesis of the Wolinella succinogenes fumarate reductase operon. *Eur J Biochem* 251**,** 418-426.

Simon, J., Sanger, M., Schuster, S.C., and Gross, R. (2003). Electron transport to periplasmic nitrate reductase (NapA) of *Wolinella succinogenes* is independent of a NapC protein. *Mol Microbiol* 49**,** 69-79.

Sousa, P.M., Videira, M.A., Santos, F.A., Hood, B.L., Conrads, T.P., and Melo, A.M. (2013). The bc:caa3 supercomplexes from the Gram positive bacterium *Bacillus subtilis* respiratory chain: a megacomplex organization? *Arch Biochem Biophys* 537**,** 153-160.

Stewart, V. (1982). Requirement of Fnr and NarL functions for nitrate reductase expression in *Escherichia coli* K-12. *Journal of Bacteriology* 151**,** 1320-1325.

Stewart, V., and Bledsoe, P.J. (2005). Fnr-, NarP- and NarL-dependent regulation of transcription initiation from the *Haemophilus influenzae* Rd *napF* (Periplasmic Nitrate Reductase). *Journal of Bacteriology* 187**,** 6928-6935.

Stewart, V., Lu, Y., and J., D.A. (2002). Periplasmic nitrate reductase (NapABC enzyme) supports anaerobic respiration by *Escherichia coli* K-12. *Journal of Bacteriology* 184**,** 1314-1323.

Thorell, H.D., Stenklo, K., Karlsson, J., and Nilsson, T. (2003). A gene cluster for chlorate metabolism in *Ideonella dechloratans*. *Appl Environ Microbiol* 69**,** 5585-5592.

Toledo-Cuevas, M., Barquera, B., Gennis, R.B., Wikstrom, M., and Garcia-Horsman, J.A. (1998). The cbb3-type cytochrome c oxidase from *Rhodobacter sphaeroides*, a proton-pumping heme-copper oxidase. *Biochim Biophys Acta* 1365**,** 421-434.

Trieber, C.A., Rothery, R.A., and Weiner, J.H. (1994). Multiple pathways of electron transfer in dimethyl sulfoxide reductase of *Escherichia coli*. *J Biol Chem* 269**,** 7103-7109.

Tseng, C.P., Albrecht, J., and Gunsalus, R.P. (1996). Effect of microaerophilic cell growth conditions on expression of the aerobic (*cyoABCDE* and *cydAB*) and anaerobic (*narGHJI*, *frdABCD*, and *dmsABC*) respiratory pathway genes in *Escherichia coli*. *Journal of Bacteriology* 178**,** 1094-1098.

Velasco, L., Mesa, S., Delgado, M.J., and Bedmar, E.J. (2001). Characterization of the *nirK* gene encoding the respiratory, Cu-containing nitrite reductase of *Bradyrhizobium japonicum*. *Biochim Biophys Acta* 1521**,** 130-134.

Vollack, K.U., Hartig, E., Korner, H., and Zumft, W.G. (1999). Multiple transcription factors of the FNR family in denitrifying *Pseudomonas stutzeri*: characterization of four fnr-like genes, regulatory responses and cognate metabolic processes. *Mol Microbiol* 31**,** 1681-1694.

Wang, H., and Gunsalus, R.P. (2000). The *nrfA* and *nirB* nitrite reductase operons in *Escherichia coli* are expressed differently in response to nitrate than to nitrite. *Journal of Bacteriology* 182**,** 5813-5822.

Winstedt, L., Yoshida, K., Fujita, Y., and Von Wachenfeldt, C. (1998). Cytochrome bd biosynthesis in *Bacillus subtilis*: characterization of the *cydABCD* operon. *J Bacteriol* 180**,** 6571-6580.

Yamamoto, I., Ujiiye, T., Ohshima, Y., and Satoh, T. (2001). Mutational analysis of regulatory cis-acting elements for the transcriptional activation of the *dmsCBA* operon in *Rhodobacter sphaeroides* f. sp. denitrificans. *Plant Cell Physiol* 42**,** 703-709.

Yoshimatsu, K., Iwasaki, T., and Fujiwara, T. (2002). Sequence and electron paramagnetic resonance analyses of nitrate reductase NarGH from a denitrifying halophilic euryarchaeote *Haloarcula marismortui*. *FEBS Lett* 516**,** 145-150.

Zargar, K., Conrad, A., Bernick, D.L., Lowe, T.M., Stolc, V., Hoeft, S., Oremland, R.S., Stolz, J., and Saltikov, C.W. (2012). ArxA, a new clade of arsenite oxidase within the DMSO reductase family of molybdenum oxidoreductases. *Environ Microbiol* 14**,** 1635-1645.

Zumft, W.G., Viebrock-Sambale, A., and Braun, C. (1990). Nitrous oxide reductase from denitrifying *Pseudomonas stutzeri*. Genes for copper-processing and properties of the deduced products, including a new member of the family of ATP/GTP-binding proteins. *Eur J Biochem* 192**,** 591-599.
