# Supplementary material for: Systematic genomic analysis reveals the complementary aerobic and anaerobic respiration capacities of the human gut microbiota
Source: Front Microbiol. 2014 Dec 5;5:674. doi: 10.3389/fmicb.2014.00674 (PMC4257093; doi:10.3389/fmicb.2014.00674)
Supplement: Supplementary file 3 [file Table3.DOCX]

**Table S3.** Genome context of the candidate genes for flavin-dependent microaerobic reductase in *F. prausnitzii* A2-165. The genes with flavin-binding domain (or domain interacting with flavin-binding protein) are shown in bold italic.

| **Pfam ID** | **Locus_tag** | **Predicted function (by genomic annotation)** | **Transmembrane segment** | **Signal peptide** |
| --- | --- | --- | --- | --- |
| ***PF01077*** | ***FAEPRAA2165_01778*** | ***FeS cluster protein*** | **+** | **+** |
| PF00578 | FAEPRAA2165_01779 | Predicted lipoprotein, TSA/AhpC family | **-** | **+** |
| ***PF04205*** | ***FAEPRAA2165_02240*** | ***Putative FMN-binding domain protein*** | **+** | **+** |
| PF04205 | FAEPRAA2165_02241 | Putative fumarate reductase flavoprotein subunit | **-** | **-** |
| PF12801 | FAEPRAA2165_02242 | Putative iron-sulfur cluster-binding protein | **+** | **-** |
| ***PF00890*** | ***FAEPRAA2165_02925*** | ***Flavocytochrome*** | **+** | **+** |
| PF07009 | FAEPRAA2165_02924 | Conserved hypothetical protein | **+** | **-** |
