# Supplementary material for: Systematic genomic analysis reveals the complementary aerobic and anaerobic respiration capacities of the human gut microbiota
Source: Front Microbiol. 2014 Dec 5;5:674. doi: 10.3389/fmicb.2014.00674 (PMC4257093; doi:10.3389/fmicb.2014.00674)
Supplement: Supplementary file 4 [file Table4.DOCX]

**Table S4.** Orthologs of candidates for flavin-dependent microaerobic reductase in genomes of various *F. prausnitzii* strains. Locus tags for orthologous genes are shown.

| ***F. prausnitzii* strain** | | | | | **Novel**  **gene name** |
| --- | --- | --- | --- | --- | --- |
| **A2-165** | **KLE1255** | **L2-6** | **M21/2** | **SL3/3** |  |
| FAEPRAA2165_01778 | HMPREF9436_00871 | FP2_23730 | FAEPRAM212_00571 | FPR_20250 | *cyfB* |
| FAEPRAA2165_01779 | HMPREF9436_00870 | FP2_23740 | FAEPRAM212_00572 | FPR_20240 | *cyfA* |
| FAEPRAA2165_02240 | HMPREF9436_00802 | - | FAEPRAM212_03497 | FPR_03420 | - |
| FAEPRAA2165_02241 | HMPREF9436_00801 | - | FAEPRAM212_03496 | FPR_03410 | - |
| FAEPRAA2165_02242 | HMPREF9436_00800 | - | FAEPRAM212_03495 | FPR_03400 | - |
| FAEPRAA2165_02925 | HMPREF9436_01082 | FP2_16180 | FAEPRAM212_00604 | FPR_19950 | *cyfF* |
| FAEPRAA2165_02924 | HMPREF9436_01081 | FP2_16190 | FAEPRAM212_00603 | FPR_19960 | *cyfE* |
