# Supplementary material for: Systematic genomic analysis reveals the complementary aerobic and anaerobic respiration capacities of the human gut microbiota
Source: Front Microbiol. 2014 Dec 5;5:674. doi: 10.3389/fmicb.2014.00674 (PMC4257093; doi:10.3389/fmicb.2014.00674)
Supplement: Supplementary file 6 [file Table6.DOCX]

**Table S6.** Differences observed in operon structures of analyzed respiratory reductases. Catalytic subunits are bold underlined (when they are known). Enzymes lacking genes for membrane components of reductase are marked by asteriks.

| **Reductase** | **Operon structure** | **Example of genome** |
| --- | --- | --- |
| Ydh | *ydh****V****YC* | *Collinsella intestinalis* DSM 13280 |
|  | *ydh****V*** ^*^ | *Gordonibacter pamelaeae 7-10-1-b* |
|  | *ydhW****V****YS* | *Faecalibacterium prausnitzii* L2-6 |
|  | *ydhX****V****YCZD* | *Sutterella wadsworthensis* 3_1_45B |
|  | *ydhTUXW****V****Y* | *Escherichia coli* K-12 MG1655 |
| Nap | *nap****A****HG* | *Gordonibacter pamelaeae 7-10-1-b* |
|  | *napED****A****BC* | *Ralstonia* sp. 5_7_47FAA |
|  | *nap****A****GHC* | *Sutterella wadsworthensis* 3_1_45B |
|  | *nap****A****GHB* | *Burkholderiales bacterium* 1_1_47 |
|  | *napFD****A****BGHBC* | *Escherichia coli* K-12 MG1655 |
|  | *napCM****A****GH* | *Bilophila wadsworthia* 3_1_6 |
|  | *nap****A****GHBFLD* | *Helicobacter canadensis* MIT 98-5491 |
| Nrf | *nrf****A****B ^*^* | *Gordonibacter pamelaeae 7-10-1-b* |
|  | *nrfH****A****IE* | *Bacteroides thetaiotaomicron* VPI-5482 |
|  | *nrfH****A****IE, nrf****A_2_*** *^*^* | *Bacteroides xylanisolvens* XB1A |
|  | *nrf****A*** *^*^* | *Sutterella wadsworthensis* 3_1_45B |
|  | *nrf****A****BCDEFG* | *Escherichia coli* K-12 MG1655 |
|  | *nrfH****A*** | *Helicobacter canadensis* MIT 98-5491 |
| Tor | *tor****A****B* | *Gordonibacter pamelaeae 7-10-1-b* |
|  | *torC****A****D, torYZ* | *Escherichia coli* K-12 MG1655 |
| Dms | *dms****A****BC* | *Escherichia coli* K-12 MG1655 |
|  | *dms****A****B ^*^* | *Clostridium asparagiforme* DSM 15981 |
|  | *dms****A****BDH* | *Gordonibacter pamelaeae 7-10-1-b* |
|  | *dms****A****BCG* | *Salmonella enterica* Typhimurium LT2 |
| Ynf | *ynf****EF****GHI* | *Escherichia coli* K-12 MG1655 |
|  | *ynf****E****GHI* | *Citrobacter youngae* ATCC 29220 |
|  | *ynf****F****GHI* | I*ovidencia rettgeri* DSM 1131 |
|  | *ynf****F*** *^*^* | *Enterobacteriaceae bacterium* 9_2_54FAA |
| Hyf | *hyfABCDEFGHIJ* | *Escherichia coli* K-12 MG1655 |
|  | *hyfBCEFGI* | *Desulfovibrio piger* ATCC 29098 |
