# Supplementary material for: Systematic genomic analysis reveals the complementary aerobic and anaerobic respiration capacities of the human gut microbiota
Source: Front Microbiol. 2014 Dec 5;5:674. doi: 10.3389/fmicb.2014.00674 (PMC4257093; doi:10.3389/fmicb.2014.00674)

Fig. S1. Phylogenetic tree for the proteins of PF00384 family. Names of previously known proteins are shown in bold. For proteins detected in this work SEED identifiers are shown (for sequences see file Sequences S1). Braches with specificities identified by analysis of the phylogenetic tree are solid, branches with specificities identified by SDP analysis are dashed. Grey arrows point to soluble subunits without membrane components.

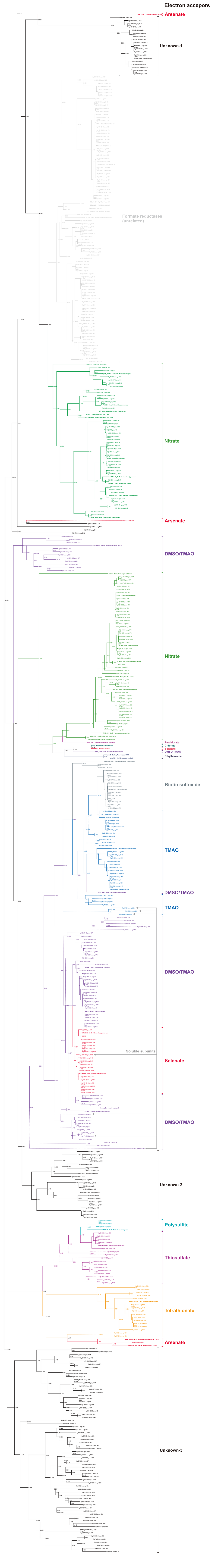

Supplement: Supplementary file 9 [file Image1.PDF]
