# Supplementary material for: Systematic genomic analysis reveals the complementary aerobic and anaerobic respiration capacities of the human gut microbiota
Source: Front Microbiol. 2014 Dec 5;5:674. doi: 10.3389/fmicb.2014.00674 (PMC4257093; doi:10.3389/fmicb.2014.00674)

Fig. S2. Phylogenetic tree for NrfA proteins found in reference genomes. Names of previously known proteins are shown in bold. For proteins detected in this work SEED identifiers are shown (for sequences see file Sequences S2). Soluble proteins existing as an additional copies to membrane complex are shown in green, soluble proteins which are present as a single NrfA copies in genome are shown in red.

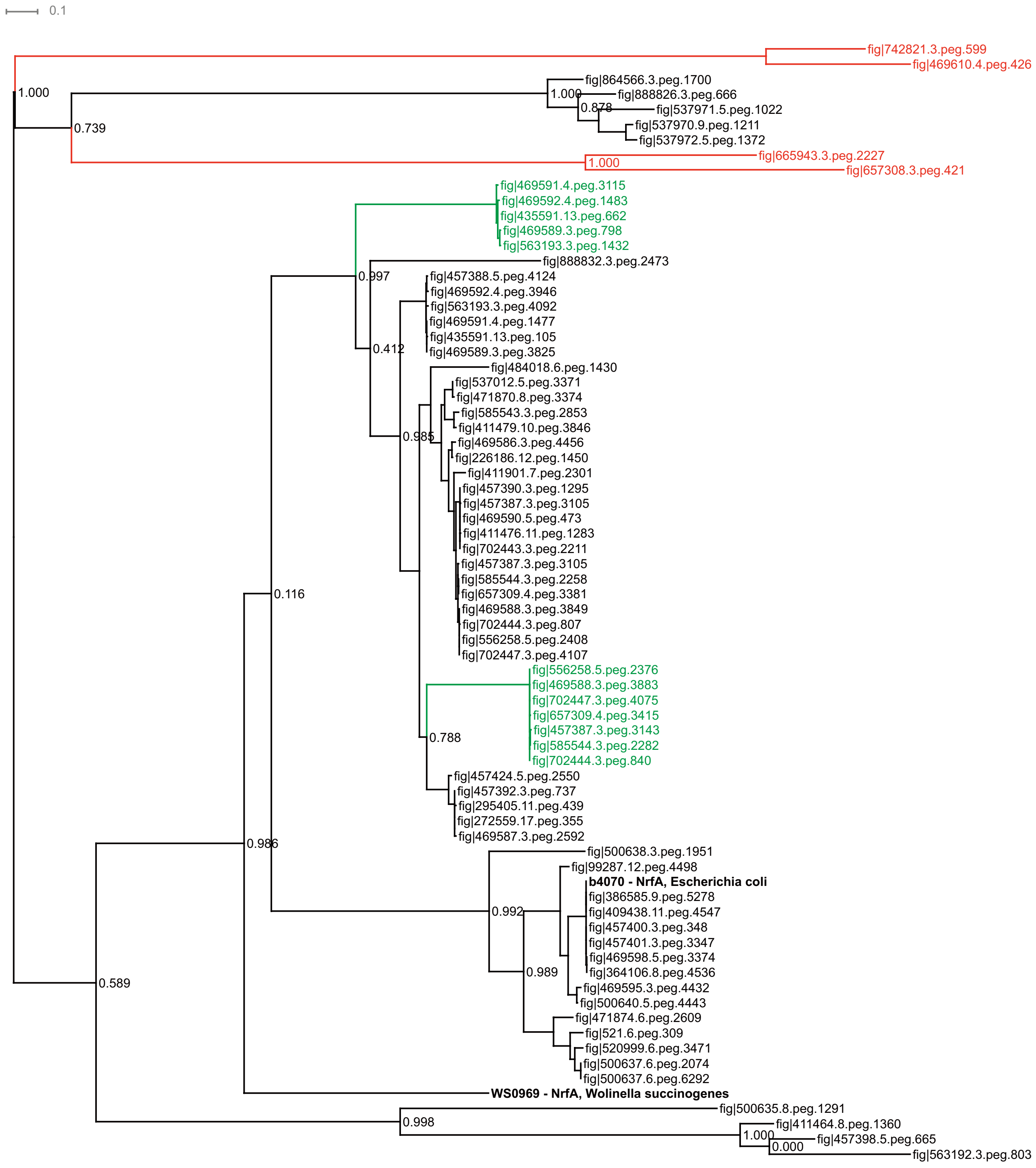

Supplement: Supplementary file 10 [file Image2.PDF]
